# Comparing the use of supervised machine learning variable selection methods in the context of two-group classification in the psychological and health sciences

**DOI:** 10.3389/fpsyg.2026.1819366

**Published:** 2026-09-07

**Authors:** Catherine M. Bain, Dingjing Shi, Yaser M. Banad, Cassandra L. Boness, Jordan E. Loeffelman

**Affiliations:** 1 Department of Psychology, California State University Northridge, Los Angeles, CA, United States; 2 Department of Psychology, University of Oklahoma, Norman, OK, United States; 3 School of Psychological and Brain Sciences, Georgia Institute of Technology, Atlanta, GA, United States; 4 School of Electrical and Computer Engineering, University of Oklahoma, Norman, OK, United States; 5 Department of Psychology, University of New Mexico, Albuquerque, NM, United States; 6 Center on Alcohol, Substance Use, and Addictions, University of New Mexico, Albuquerque, NM, United States

**Keywords:** classification, machine learning, metaheuristics, regularization, variable selection

## Abstract

**Introduction:**

Variable selection (VS) is crucial for building accurate and generalizable classification models. Reducing the necessary number of variables improves model efficiency, interpretability, and generalizability while reducing data collection burden. Despite the availability of various VS methods, their comparative performance within machine learning frameworks for classification remains unclear.

**Methods:**

This study conducted a large-scale Monte Carlo simulation to compare VS methods from distinct families: regularization techniques (LASSO, Elastic Net), tree-based methods (random forest-based Boruta wrapper), penalized support vector machines, and metaheuristics (genetic algorithm). An empirical example from an alcohol use disorder dataset illustrated the comparative findings. Supplementary simulations evaluated four additional filter methods, re-estimated performance under stratified 10-fold cross-validation, and quantified overfitting via train-test performance gaps.

**Results:**

In the main simulation, the genetic algorithm demonstrated the strongest overall classification accuracy, while LASSO offered the most parsimonious solution with minimal performance loss. All three top-performing methods (GA, LASSO, Elastic Net) substantially reduced the number of variables from the saturated model. Supplementary analyses aligned with main simulation findings.

**Discussion:**

These findings highlight the practical value of machine learning VS methods in psychological research by demonstrating how to balance accuracy, interpretability, and scalability. The results provide guidance for researchers selecting among competing VS approaches based on their priorities regarding model performance versus parsimony.

## Introduction

The volume of data available to behavioral scientists has grown exponentially (Chen and Wojcik, 2016; Foster et al., 2020; Harlow and Oswald, 2016). These datasets often involve large samples, numerous predictors, or both. Including all possible predictors is inefficient, non-parsimonious, and sometimes infeasible. As such, variable selection (VS) plays a crucial role in enhancing the reproducibility and generalizability of findings (Heinze et al., 2018; Steiner et al., 2010; Yarkoni and Westfall, 2017). Traditional VS methods like forward, backward, and stepwise regression produce suboptimal results; failing to distinguish signal from noise, underestimating *p*-values, and impeding replication of results (Derksen and Keselman, 1992; Thompson, 1995; Whittingham et al., 2006; Wiegand, 2010). These issues raise concerns about model overfitting and the credibility of conclusions.

Two-group classification is common in behavioral science research. For example, in clinical contexts, models help identify individuals at risk of mental health disorders. As such, researchers have developed a variety of VS approaches specifically for classification contexts (e.g., Brusco et al., 2019; El Haouij et al., 2018; Fouskakis and Draper, 2008; Hafeez et al., 2021; Pacheco et al., 2009; Speiser et al., 2019); most frequently, researchers turn to logistic regression-based techniques like the least absolute shrinkage and selection operator (LASSO) and Elastic Net (EN). More complex models like random forests (RF) or support vector machines (SVM) are available, but less commonly used.

Although prior research has compared VS methods, most focus within methodological families (e.g., regularization vs. regularization; Sadiq and Shah, 2025; Waldmann et al., 2013; Yoo and Rho, 2022; e.g., tree vs. tree; Hu and Li, 2022; Müller et al., 2026). Few have compared across families (e.g., RF vs. regularization; Ahmed et al., 2013; Cutler et al., 2007; Holden et al., 2011; Minari et al., 2025; Sen et al., 2023; Vey et al., 2026). Lastly, some published studies simply use multiple methods across a variety of families, but fail to compare their results (e.g., Mǎnescu and Mǎnescu, 2025). Given the increasing complexity of psychological and health data, cross-family evaluations are critical for guiding method selection.

The purpose of the present study is to provide applied researchers in the psychological and health sciences with direct, head-to-head evidence on how supervised machine learning VS methods from distinct methodological families—regularization (embedded), tree-based, and metaheuristic—perform for two-group classification, and to characterize how that performance depends on data characteristics such as sample size, number of predictors, class prevalence, and noise. Concretely, we ask: (1) which methods achieve the best out-of-sample classification performance and variable selection accuracy across realistic data conditions, and (2) how methods trade off predictive performance against parsimony and computational cost. A separate set of supplementary simulations extend this comparison to a filter family of methods, to stratified cross-validation, and to an explicit overfitting analysis (see Supplementary materials).

This study addresses a gap in current research by comparing VS techniques from three methodological families: regularization (LASSO, EN, an implementation of an SVM), tree-based (RF, via the Boruta wrapper), and metaheuristics (Genetic Algorithm; GA). Our findings aim to inform behavioral researchers about the strengths and limitations of each approach, informed by data characteristics and specific research questions. The paper is organized as follows. We begin by introducing the VS techniques. Next, we compare their performance in a large-scale Monte Carlo simulation study, provide evidence from additional supplemental simulations, and provide an empirical example of alcohol use disorder (AUD). Finally, we discuss key findings and implications for future research.

## Comparison of variable selection techniques in classification

The study compares the performance of GA, LASSO, EN, and implementations of SVM and RF as VS techniques in the context of two-group classification. These methods were chosen for their popularity, versatility, and demonstrated effectiveness in a wide range of fields (e.g., Bacchetti, 2010; Bainter et al., 2023; Boness et al., 2019; Chen and Wojcik, 2016; Cutler et al., 2007; Eisenbarth et al., 2015; Fife and D'Onofrio, 2023; Fouskakis and Draper, 2008). LASSO and EN, widely used regularization techniques, are renowned for their ability to produce sparse models by shrinking regression coefficients toward zero, effectively performing VS (Bainter et al., 2023; Kok et al., 2021; McNeish, 2015). The GA, a metaheuristic approach, offers a powerful search strategy for identifying optimal variable subsets based on a user-defined objective function. RF, an ensemble learning method based on decision trees, is known for its robust predictive performance and its ability to provide inherent measures of variable importance. Finally, SVM, a powerful classification algorithm, can be adapted for VS by incorporating penalized approaches. Other VS methods exist (e.g., Bayesian methods; Shi et al., 2023), but were excluded due to computational demands and the requirement of specialized expertise, potentially limiting their accessibility for many researchers in the psychological sciences. A filter family of models (mutual information, variance threshold, Pearson correlation, and χ^2^ screening) are evaluated in the supplementary simulations to complete the filter–embedded–wrapper–metaheuristic framework (see Supplementary materials).

We anticipated performance differences based on various manipulated data characteristics. Specifically, LASSO and EN were expected to underperform when the data contained more predictors than observations (i.e., *p* > *n*) due to degree of freedom constraints (Tibshirani and Taylor, 2012; Zou and Hastie, 2005; Zou et al., 2007). GA was expected to excel in predictive accuracy due to its optimization-driven design and adaptability across diverse data structures (Brusco et al., 2019; Eisenbarth et al., 2015; Goldberg, 1989; Taha et al., 2025).

## Review of selected VS techniques in classification

### Regularization techniques

Regularization techniques address VS by adding a penalty to the loss function, balancing model complexity and predictive performance. The general regularized logistic regression function is:


$$\begin{array}{l} L^{Reg}\left(\beta \right)=L^{MLE}\left(\beta \right)-\lambda ℘\left(\beta \right) \end{array}$$ (1)

where, *L*^*MLE*^ is the standard maximum likelihood estimate (MLE) optimization function, λ is a tuning parameter controlling the penalty strength, and ℘(β) varies by technique. Cross-validation or a grid search can be used to determine the appropriate value for the λ parameter, which is vitally important as the function must be penalized enough to reduce overfitting but not too much as to render meaningless estimates (McNeish, 2015). Cross-validation is most often recommended and involves partitioning the data into training and validation sets to identify the tuning parameter that minimizes the prediction error on the validation set.

### LASSO

In classification contexts, LASSO (Tibshirani, 1996) uses an L1 penalty that shrinks some coefficients of the logistic regression equation (Equation 1) to exactly zero, yielding sparse, interpretable models. It is particularly effective for selecting relevant predictors and improving generalizability (Descloux and Sardy, 2021; Kok et al., 2021; McNeish, 2015). Despite its advantages, LASSO is limited in that it cannot select more than *n* predictors when *p* > *n* and struggles with highly correlated predictors, often arbitrarily selecting one among them (Zou and Hastie, 2005). Its adoption in behavioral sciences remains limited, despite its implementation in major software platforms, with some researchers going so far as to argue that LASSO is not implemented enough within the behavioral sciences (Johnson and Sinharay, 2011; McNeish, 2015).

### Elastic Net

EN (Zou and Hastie, 2005) combines the L1 (LASSO) and the L2 penalties—the L2 penalty being the sum of squared coefficients used by ridge regression—overcoming LASSO's limitations with correlated variables and enabling the selection of all *p* predictors, even when *p* > *n*. Each penalty has unique hyperparameters (λ_1_ and λ_2_), allowing for differential weighting of the penalties. EN allows for the selection of multiple variables from a set with strong pairwise correlations and often outperforms LASSO in classification accuracy and model parsimony (Algamal and Lee, 2015; Liu and Li, 2017). It has been shown to reduce dimensionality dramatically while preserving key predictors (Marafino et al., 2015). However, clinical research has shown that EN is limited in coefficient estimations of variables with similar coefficients (Algamal and Lee, 2015).

### Tree-based methods (random forest and the Boruta wrapper)

**Figure 1:**
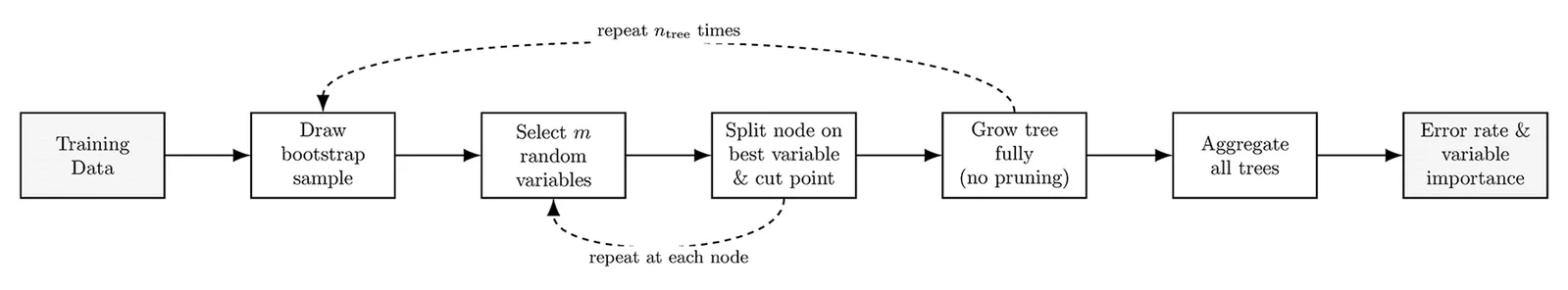
A flowchart for the random forests algorithm representing a workflow for classification problems.

RF (Breiman, 2001) is an ensemble method that builds multiple classification trees using bootstrap samples. Final classifications are determined by aggregating across trees (e.g., majority vote). RF provides built-in variable importance metrics, making it a practical tool for VS (Speiser et al., 2015). RF is advantageous due to its capacity to handle mixed data types, interaction effects, and small-sample, high-dimensional settings. It is resistant to overfitting and is widely supported in common software packages. RF has demonstrated superior performance in prediction accuracy across multiple domains (Fernandez-Delgado et al., 2014), and its adoption in psychological research is growing (Ammerman et al., 2018; El Haouij et al., 2018; Fife and D'Onofrio, 2023; Wallert et al., 2018). The general process of building a RF model can be found in Figure 1.

Boruta (Kursa and Rudnicki, 2010) is not a stand-alone classifier but a wrapper feature-selection method built around RF; we evaluate it here as the tree-based wrapper representative. Throughout the tables and figures, “Boruta” therefore denotes the RF-based wrapper selection procedure rather than a distinct model family.

### Boruta

Boruta (Kursa and Rudnicki, 2010) is a wrapper method built around RF. It creates permuted “shadow” variables by randomly permuting the values of input variables, fits an RF model, and then ranks variables by comparing real importance to shadow importance scores. If a variable is significantly outperforming its shadow counterpart (using a *z*-test), then it is marked as “confirmed” important. If a feature score is significantly worse, it is marked as “rejected”. The remaining features are marked “tentatively confirmed”.^1^ This process iterates until all variables are classified or a set iteration limit is reached. Boruta is valued for its ability to eliminate irrelevant features while retaining potentially relevant ones, particularly in complex datasets.

### Support vector machines (SVM)

SVMs (Cortes and Vapnik, 1995) are powerful classifiers that identify a hyperplane separating classes with the widest possible margin. While popular in neuroscience research (Javed et al., 2021; Orrù et al., 2012; Sánchez-Reolid et al., 2020; Yang et al., 2017), SVMs remain underutilized in other psychological subfields (Yang et al., 2024). This study employs soft-margin SVM (as compared to a hard margin) and a radial basis kernel to handle non-linear boundaries. Soft margins allow for a controlled degree of misclassification, enhancing model generalizability.

### Penalized SVM

Traditional SVMs do not perform VS; however, penalized variants use penalties like LASSO, EN, or the smoothly clipped absolute deviation (SCAD) penalty (Becker et al., 2009) to induce sparsity. SCAD is a non-convex penalty that, unlike the L1 penalty, applies little or no shrinkage to large coefficients while still shrinking small ones to zero, which reduces the estimation bias L1 imposes on strong predictors. This study applies a hybrid EN-SCAD penalty (Becker et al., 2011), which balances SCAD's strictness with EN's flexibility, optimizing selection in non-sparse settings. For more on this approach, see Becker et al. (2011).

### Metaheuristic approach (genetic algorithm)

**Figure 2:**
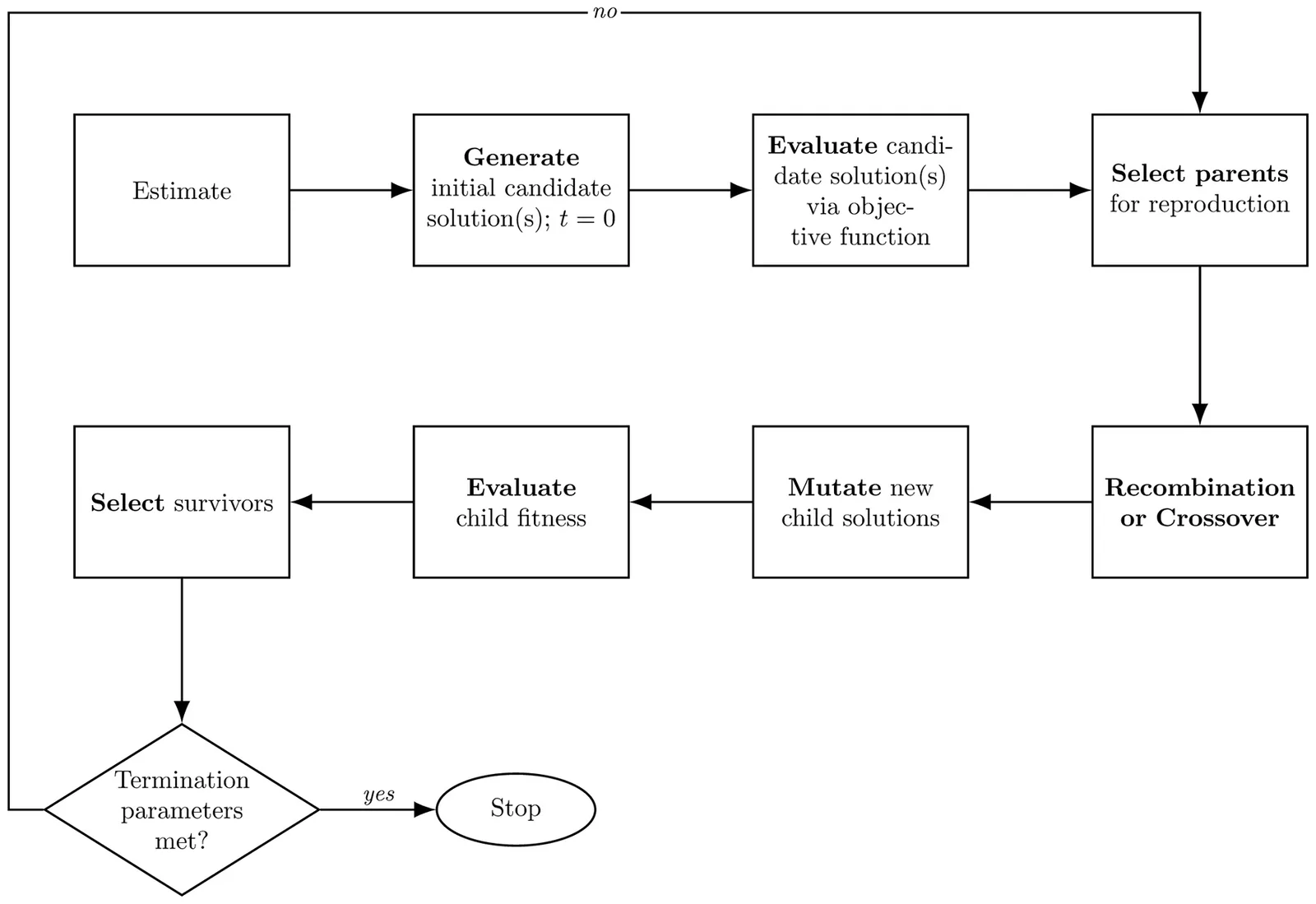
The basic algorithmic steps of the genetic algorithm.

GAs (Goldberg, 1989; Holland, 1992) simulate natural selection to search for optimal variable subsets. The GA implemented in this paper does have a set classification model (as is seen in Koehler, 1991; Pendharkar and Nanda, 2006), but rather focuses on searching through possible combinations of variables (similar to the algorithms seen in Brusco et al., 2019; Pacheco et al., 2006). The full algorithm (Figure 2) iteratively evolves a population of candidate solutions, recursively improving the solution through crossover and mutation until it reaches a subset that optimizes the selected criteria (e.g., a balance between predictive ability and subset size). Each chromosome represents a subset of predictors. Fitness is evaluated using an optimization criterion—in this case, the adjusted rand index (ARI; Hubert and Arabie, 1985), comparing predicted and true classifications.^2^ The GA implementation used here (Eisenbarth et al., 2015) uses logistic regression to predict classifications.

The GA is robust to changes in the data and is advantageous because it considers a population of solutions rather than a single solution, investigating more possible solutions than other VS methods. GAs have been shown to perform well for various psychological optimization tasks, including scale construction and multidimensional scaling (Eisenbarth et al., 2015; Rachmani et al., 2019; Sahdra et al., 2016; Schroeders et al., 2016; Yarkoni, 2010).

Because the GA implemented here pairs its evolutionary search with a single internal classifier (logistic regression), the present design cannot by itself separate the contribution of the search

strategy from that of the fixed linear model. We take up this question directly in a companion study (Bain and Shi, 2026), which holds the GA search strategy constant while fully crossing three internal classifiers (support vector machine, *k*-nearest neighbors, and logistic regression) with three fitness functions. Its central finding is directly relevant here: the internal classifier had an effect on out-of-sample classification performance and computational cost but had essentially no effect on which variables the search returned (subset recovery and size were nearly invariant across classifiers). This indicates that what drives GA variable selection is the fitness function rather than the internal model. We therefore retain logistic regression as the GA base learner in the present study and refer readers to the companion paper for the internal-model comparison; we return to this point in the Discussion.

### Brief summary

Each technique offers distinct advantages and trade-offs. Regularization methods yield sparse, interpretable models but vary in handling multicollinearity and dimensionality constraints. Tree-based methods are powerful and flexible, with built-in mechanisms for evaluating variable importance. SVMs, especially with penalized adaptations, provide robust classification and selection capabilities in high-dimensional contexts. GAs offer a global optimization perspective, especially useful when the predictor space is vast and heterogeneous. These methods are increasingly relevant for behavioral and psychological science, where large, complex datasets demand robust and interpretable modeling strategies. Selecting the appropriate technique depends on data characteristics, theoretical goals, and computational resources.

## A simulation study

We conducted a Monte Carlo simulation to systematically compare VS techniques (listed in Table 1) for two-group classification tasks. Models were evaluated under two complementary sets of simulations. In the primary set of simulations, each method was trained on 70% of the data and evaluated on the remaining 30% using a single stratified hold-out split. However, a single split can be noisy, especially at small sample sizes, so using a focused subset of the simulation design, we used a stratified 10-fold cross-validation. We also used this subset of conditions to add methods from the filter family and a comparison between train and test data outcomes to explicitly assess overfitting. We present the hold-out analysis as the main simulation and the set of simulations with filter methods and cross validation as an integrated robustness and scope layer, reporting the key results of these simulations in the subsections below and its full tables in the Supplementary materials.

**Table 1:** Summary of variable selection methods.

Family	Method (abbreviation)	R package (citation)
Metaheuristic	Genetic Algorithm (GA)^a^	*GA* (Scrucca, 2013)
Regularization (embedded)	LASSO	*glmnet* (Friedman et al., 2010)
	Elastic Net (EN)
Penalized SVM	Elastic SVM (eSVM)	*penalizedSVM* (Becker et al., 2011)
Tree-based wrapper	Boruta (RF-based wrapper)	Boruta (Kursa and Rudnicki, 2010)
Filter	Mutual information (MI)	Base R
	Variance threshold	
	Pearson correlation
	χ^2^

Grid-tuned LASSO and EN variants performed worse than their cross-validated counterparts and are not discussed further. Boruta denotes the random forest (RF) based wrapper selection procedure, not a stand-alone classifier.

^a^The GA implementation uses an internal logistic regression classifier; the companion study (Bain and Shi, 2026) shows that the internal classifier changes predictive performance and cost but not the selected variable set, so the GA selection results are unlikely to be an artifact of the logistic base learner. The filter methods are evaluated in the cross-validated re-analysis (Supplementary materials).

Table 1 lists the methods and their software implementations, organized by the filter, embedded, wrapper, and metaheuristic families. The filter family (mutual information, variance threshold, Pearson correlation, and χ^2^ screening) was added to increase the scope of the taxonomy and to provide a cost-efficient screening baseline. Each filter ranks candidate predictors by a univariate association statistic, retains the top 50%, and passes the retained set to a logistic classifier, placing the filters in the same evaluation framework as other families.

### Data generating conditions

The simulation followed design principles consistent with prior literature (Brusco, 2014; Jiang et al., 2021; Loeffelman et al., 2020). In the main simulation, each data condition was replicated 1,000 times to ensure stability of results. Data generation varied across five key features: predictor type, prevalence rate, number of predictors, sample size, and signal-to-noise ratio.

### Distribution of the predictors

Predictor variables were either continuous or binary. Continuous predictors were drawn from a multivariate normal distribution with a mean of zero and covariances ranging from 0.03 to 0.60. Binary predictors were obtained by dichotomizing the continuous predictors described in the preceding sentence at a fixed threshold on the latent normal scale (a median split at the variable's mean), so that each binary predictor is a thresholded version of its continuous counterpart. This design allowed for consistent comparison across both variable types while controlling for underlying distributional properties.

### Prevalence rates (proportion of positive cases)

Outcome variables were generated using the same distributional structure as the predictors and then transformed into binary outcomes to simulate a range of prevalence rates. Specifically, probabilities of class membership [i.e., *P*(*y* = 1∣*X*)] were set at 0.20, 0.25, 0.30, and 0.40. These base rates were chosen to reflect diagnostic prevalences commonly reported for disorders defined by the Diagnostic and Statistical Manual of Mental Disorders, Fifth Edition (DSM-5; American Psychiatric Association, 2020), which is also the diagnostic standard used to define the outcome in the empirical example (see Empirical Example); we note the DSM-5 basis here, at first mention, rather than only in the applied section. These thresholds allowed us to assess classifier performance under conditions of imbalanced group sizes.

### Number of predictors and sample size

The number of predictor variables varied across conditions, with datasets containing either 10, 50, or 100 predictors. Sample size was also manipulated, with data sets simulated to include 50, 200, 500, or 1,000 observations. Prior studies offer mixed recommendations about adequate sample sizes for machine learning techniques. Some previous literature indicates that a sample size of 200 is adequate to see strong performance in random forest (Bacchetti, 2010) while other researchers have indicated a much larger sample size is needed (Floares et al., 2017). One study found that a sample size of 200 is adequate for SVM and logistic regression models (Entezari-Maleki et al., 2009), though it does note that the stability of performance increases as sample size increases.

### Signal to noise ratio

To simulate realistic classification challenges, each dataset also varied in terms of signal-to-noise ratio. This was done by including either no noise variables, a number of noise variables equal to the total number of predictors, or half that amount. Noise variables were generated using the same distributional parameters as the signal variables but were explicitly uncorrelated with the outcome variable. This setup allowed us to test each method's ability to effectively distinguish relevant predictors from irrelevant ones.

### Analytical techniques, outcomes of interest, and evaluation

After the datasets were generated, the columns of each dataset were randomly permuted, while retaining the indices of the true signal variables. Altogether, the combination of manipulated factors resulted in 288 unique simulation conditions in the main simulation.

Each model's performance was assessed using six standard classification metrics calculated on the held-out test data. These included prediction error rate (PER), the area under the receiver operating characteristic curve (AUC), Cohen's kappa (Cohen, 1960), sensitivity, specificity, and the *F*1 score. Higher AUC, kappa, sensitivity, specificity, and F1 indicate better performance, and lower PER and false positive rate (FPR) indicate better performance. We also recorded the proportion of variables selected and the FPR (the proportion of selected variables that were noise), offering insights into the sparsity and parsimony of the models. A lower FPR is considered better, and a small proportion of variables indicates a more parsimonious model.

To allow a fair comparison of overfitting risk across methods of differing capacity, in the supplementary simulations, each of the six classification metrics was also computed on the training folds, and a train–test gap (training metric minus cross-validated test metric) was recorded for every metric. Larger positive gaps indicate greater optimism (i.e., more overfitting). These train-set outputs and gap statistics are reported in a dedicated results subsection (Overfitting Analysis) and in the Supplementary materials, so that high-capacity methods (e.g., GA, Boruta) and sparser methods (e.g., LASSO, EN, filters) can be compared on the difference between training and held-out performance rather than on held-out performance alone.

### Effect sizes for performance metrics

To evaluate the influence of each experimental factor and method on model performance, a series of analyses of variance (ANOVAs) models were conducted for each outcome. Main effects and interactions up to two-way interactions were included in the analysis. To ensure that the most influential effects were of focus, we probed all effect sizes (partial η^2^*s*) greater than or equal to 0.06 (medium effect; Cohen, 2009). All other effects were omitted.

### Simulation results

Overall distributions of all outcomes grouped by predictor type and classification model can be found in Table 2. Given the 288 unique data conditions, we present a subset of results that focuses on key trends and theoretically meaningful interactions. The selection of conditions was guided by the magnitude and consistency of observed effects, with an emphasis on at least medium effect sizes (η^2^ > 0.06; Cohen, 2009) and interactions that exhibited divergent method performance. The results are organized by outcome of interest. Results from all ANOVA models can be found in Table 3, and the cross-validated effect sizes from the supplementary simulations are in Supplementary Table S4. All simulation code and results are archived on OSF (https://osf.io/qysh4/?view_only=85146f45202f4c9f944ee1d7622954b3).

**Table 2:** Distribution of all outcomes for all methods.

Method	*N* invalid	PER	AUC	Kappa	Sensitivity	Specificity	*F*1 score	# Selected	FPR
		Mean (SD)	Mean (SD)	Mean (SD)	Mean (SD)	Mean (SD)	Mean (SD)	Mean (SD)	Mean (SD)
Binary predictors									
Boruta	5,383	0.26 (0.1)	0.70 (0.14)	0.25 (0.25)	0.89 (0.10)	0.35 (0.28)	0.83 (0.08)	0.36 (0.26)	0.02 (0.10)
eSVM	78,749	0.28 (0.1)	0.56 (0.11)	0.11 (0.21)	0.96 (0.10)	0.15 (0.28)	0.82 (0.08)	0.46 (0.34)	0.15 (0.25)
Elastic Net	401	0.28 (0.1)	0.63 (0.16)	0.04 (0.14)	0.99 (0.03)	0.04 (0.14)	0.83 (0.07)	0.27 (0.22)	0.05 (0.13)
GA	30	0.11 (0.1)	0.89 (0.12)	0.67 (0.29)	0.96 (0.07)	0.70 (0.29)	0.92 (0.07)	0.58 (0.10)	0.27 (0.21)
LASSO	415	0.28 (0.1)	0.62 (0.15)	0.04 (0.14)	0.99 (0.04)	0.05 (0.15)	0.83 (0.07)	0.18 (0.18)	0.05 (0.16)
Continuous predictors									
Boruta	5,589	0.28 (0.11)	0.68 (0.13)	0.20 (0.22)	0.87 (0.12)	0.31 (0.24)	0.81 (0.10)	0.42 (0.3)	0.04 (0.15)
eSVM	35,607	0.27 (0.11)	0.56 (0.10)	0.13 (0.21)	0.95 (0.10)	0.17 (0.25)	0.82 (0.09)	0.53 (0.30)	0.27 (0.24)
Elastic Net	1,069	0.28 (0.10)	0.62 (0.15)	0.05 (0.11)	0.99 (0.03)	0.05 (0.11)	0.83 (0.07)	0.30 (0.22)	0.07 (0.17)
GA	686	0.10 (0.11)	0.91 (0.12)	0.71 (0.33)	0.96 (0.06)	0.74 (0.31)	0.93 (0.08)	0.56 (0.10)	0.28 (0.21)
LASSO	1,091	0.28 (0.10)	0.62 (0.15)	0.05 (0.11)	0.99 (0.04)	0.05 (0.11)	0.83 (0.07)	0.13 (0.14)	0.08 (0.21)

SD = standard deviation; eSVM = elastic net SVM; PER, prediction error rate; AUC, area under the curve; # selected = proportion of total variables that were selected; FPR = proportion of selected variables that were noise variables. For comparison, higher AUC, kappa, sensitivity, specificity, and *F*1 score were considered better. Lower PER, Type I, and Type II errors were considered better. The number of invalid models indicates the number of models that either did not converge or did not select at least one variable. A total of 144,000 models were possible for each method within each predictor type.

**Table 3:** Effect sizes: main effects and interactions of all outcome metrics.

Sim. Factor	Partial η^2^							
	PER	AUC	Kappa	Sensitivity	Specificity	*F*1 score	# Selected	FPR
Predictor type	–	–	–	–	–	–	–	–
Rate	**0.27**	–	–	**0.11**	–	**0.33**	–	–
# Preds	–	–	–	–	–	–	0.13	–
*N*	–	–	–	–	–	–	0.09	–
Noise	–	–	–	–	–	–	–	0.79
PN	–	–	–	–	–	–	–	–
Method	0.33	**0.30**	0.44	0.36	**0.43**	0.28	0.84	0.54
# Preds × method	0.07	–	0.06	–	–	–	0.16	–
*N* × method	0.08	–	0.07	0.11	–	–	0.21	–
Noise × method	–	–	–	–	–	–	0.08	0.40
PN × method	–	–	–	–	–	0.08	0.14	0.10

Rate = prevalence rate of the diagnosed (or 1) class; noise = proportion of predictors that are noise; PN = if the dataset has more predictors than observations; # selected = proportion of total variables that were selected; FPR = proportion of selected variables that were noise variables; PER, prediction error rate; AUC, area under the curve. Effect sizes < 0.06 (medium effect; Cohen, 2009) were omitted. Bolded values indicate influential main effects that are not part of an influential interaction effect. A model with only main effects was first examined for each outcome. We then probed all possible two- and three-way interactions that contained at least one variable with an influential main effect. In such cases when a main effect or interaction was not found to be influential on any classification measure, it was omitted from this table. Effects were determined to be influential if they met or exceeded 0.06 (medium effect; Cohen, 2009).

### Effects of data characteristics

**Figure 3:**
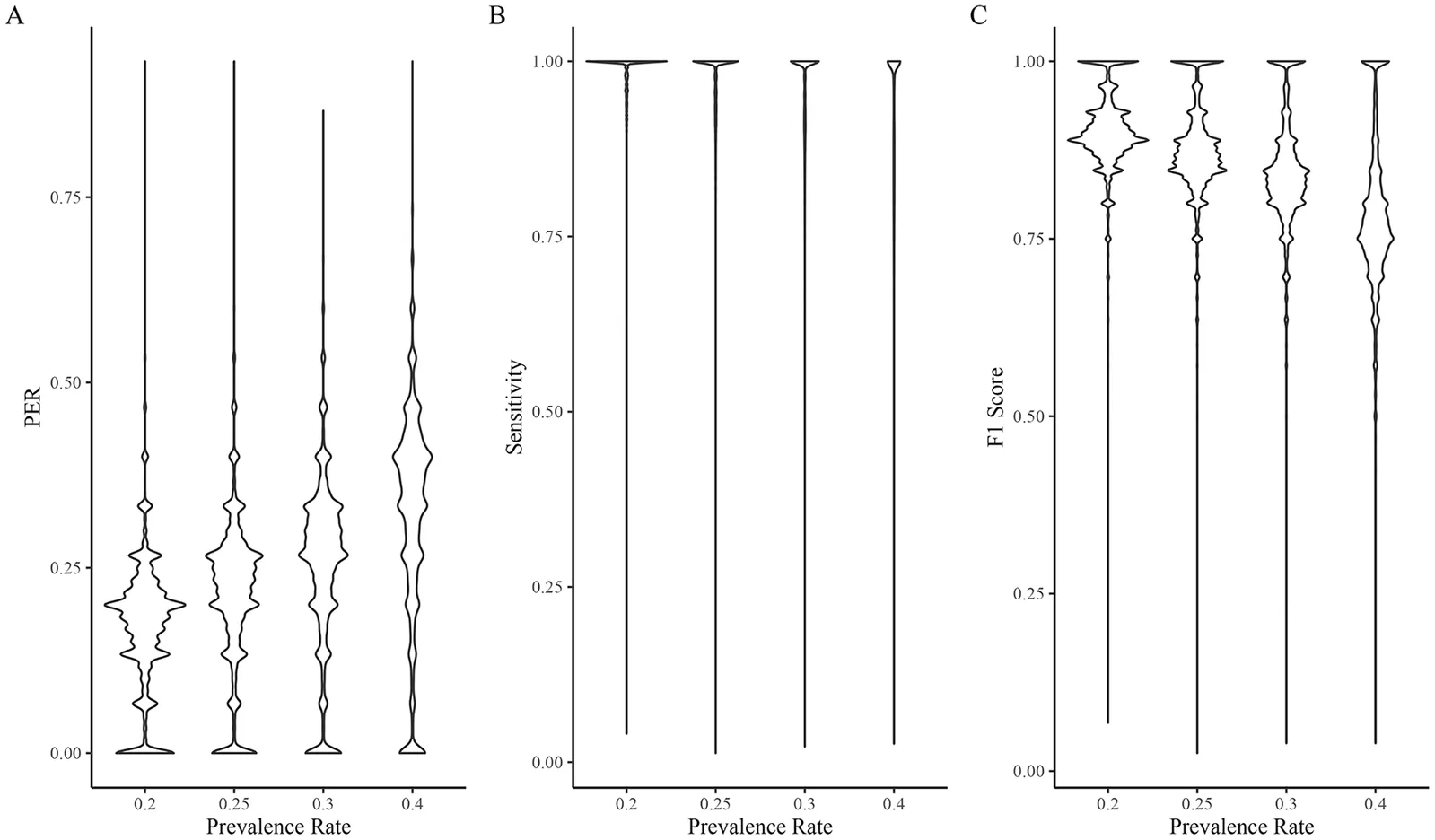
Effects of data characteristics. PER, prediction error rate. Panel **(A)** displays the main effect of prevalence rate (or the proportion of the sample in class 1) on PER. Panel **(B)** displays the main effect of prevalence rate on sensitivity. Panel **(C)** displays the main effect of the prevalence rate on the *F*1 score.

We first examined the influential effects of the data characteristics, as presented in Figure 3 and Table 3. Notably, only the effect of prevalence rate (or the balance in class sizes) was found to be an influential main effect. Specifically, we found that higher prevalence rates, or more balanced class sizes, led to higher prediction error rates (Figure 3A; η^2^ = 0.27), lower sensitivity (Figure 3B; η^2^ = 0.11), and lower *F*1 scores (Figure 3C; η^2^ = 0.33). This effect did not vary across classification methods. This trend may reflect the fact that many machine learning models are implicitly optimized for majority class performance, and as such, they may perform better when class distributions are skewed. When data become more balanced, the model is required to perform equally well on both classes, revealing potential weaknesses in its ability to generalize across underrepresented cases.

### Effects of method choice on classification performance

We now shift our focus to the effects resulting from the method (Boruta, EN, Elastic SVM, GA, and LASSO) on each outcome of interest.

### PER

**Figure 4:**
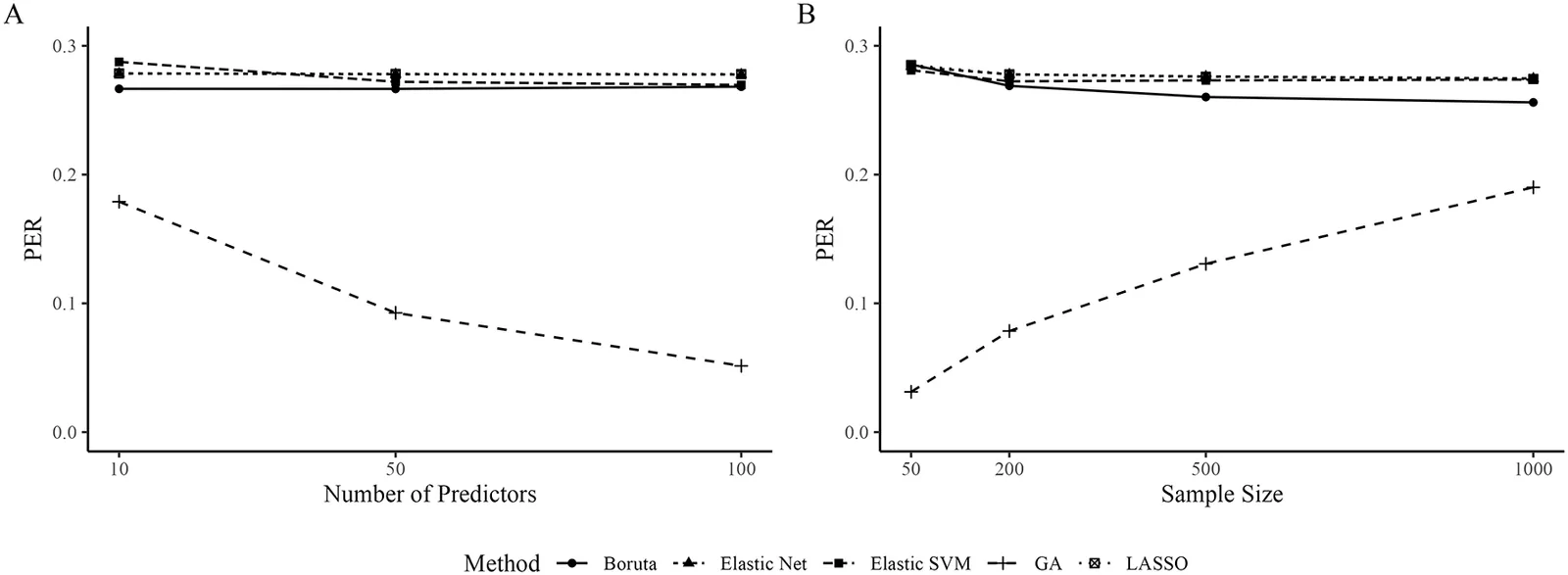
Comparison of PER between methods across different conditions. PER, prediction error rate; Panel **(A)** displays the interaction of the number of predictors and the method on the PER. Panel **(B)** displays the interaction of the sample size and the method on the PER. The performance of LASSO and Elastic Net models is so similar that they overlap completely in both Panels **(A, B)**.

The number of predictors did not influence the PER of EN or LASSO models, but did influence the PER of Boruta, GA, and Elastic SVM models (Figure 4A; η^2^ = 0.07). The PER of Boruta models increased slightly as the number of predictors increased. The PER of both GA models and Elastic SVM models decreased as the number of predictors increased. This relationship was strongest for GA models. The sample size influenced the PER of all models (Figure 4B; η^2^ = 0.08). The PER of Boruta, LASSO, EN, and Elastic SVM models saw slight decreases as sample sizes increased. The PER of GA models increased as the sample size increased. However, the average PER of GA models was lower than the PER of all other models across all numbers of predictors and all sample sizes.

### AUC and F1 score

**Figure 5:**
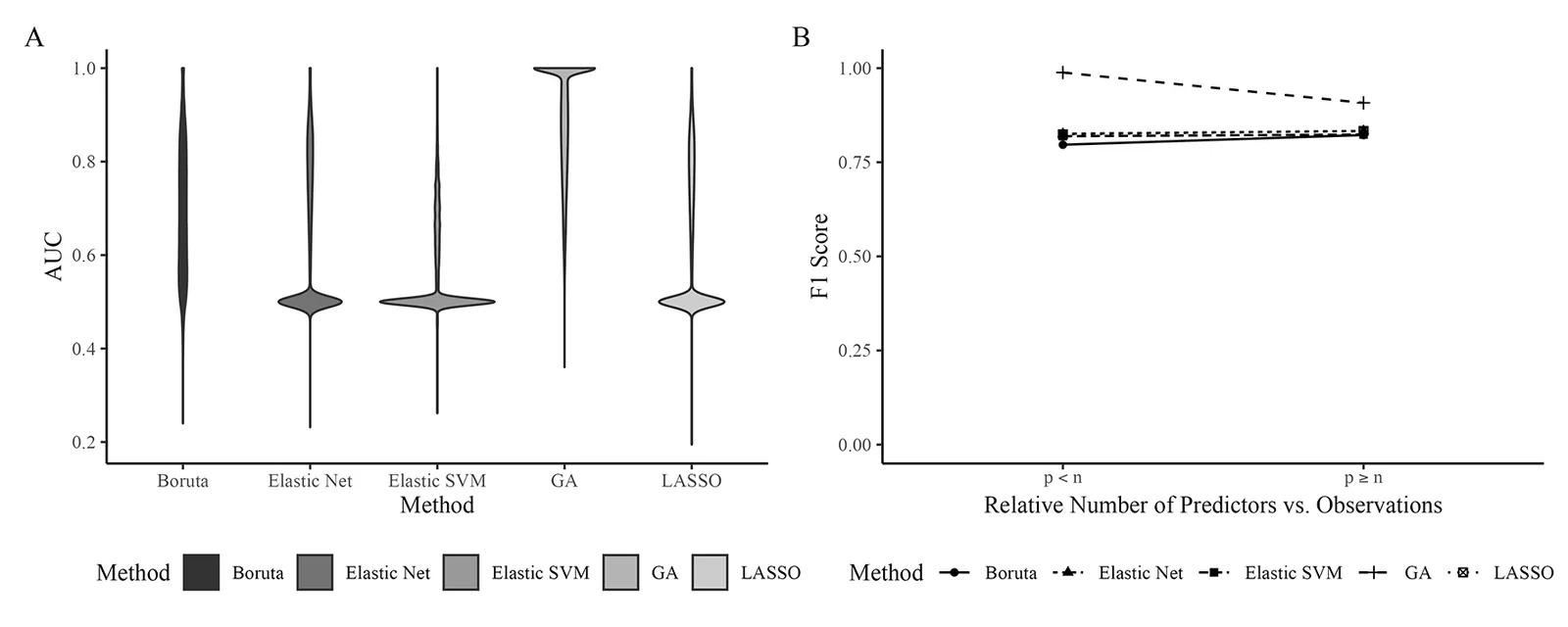
Comparison of AUC and *F*1 scores between methods across different conditions. Panel **(A)** displays the main effect method on the AUC. Panel **(B)** displays the interaction of the ratio of predictors to observations and the method on the *F*1 Score. The performance of LASSO and Elastic Net models is so similar that they overlap completely in Panel **(B)**.

Only the method was found to be meaningful on the AUC of models (Figure 5A; η^2^ = 0.30). Specifically, the AUC of GA models was found to be highest on average, with Boruta models having the second-highest average AUC. Average AUCs of EN, Elastic SVM, and LASSO models were all comparable. The AUC of Boruta models was found to be most variable across replications and data conditions. The AUC of GA models was found to be the least variable across replications and data conditions. Regarding *F*1 Score, if the data contained fewer predictors than observations (*p*<*n*), the GA performed better than in other predictor to observation ratios (Figure 5B; 0.08). All other methods performed slightly worse when the data contained fewer predictors than observations (*p*<*n*) than in other predictor to observation ratios. This finding is interesting, given degree of freedom constraints on regression-based approaches.

### Kappa

**Figure 6:**
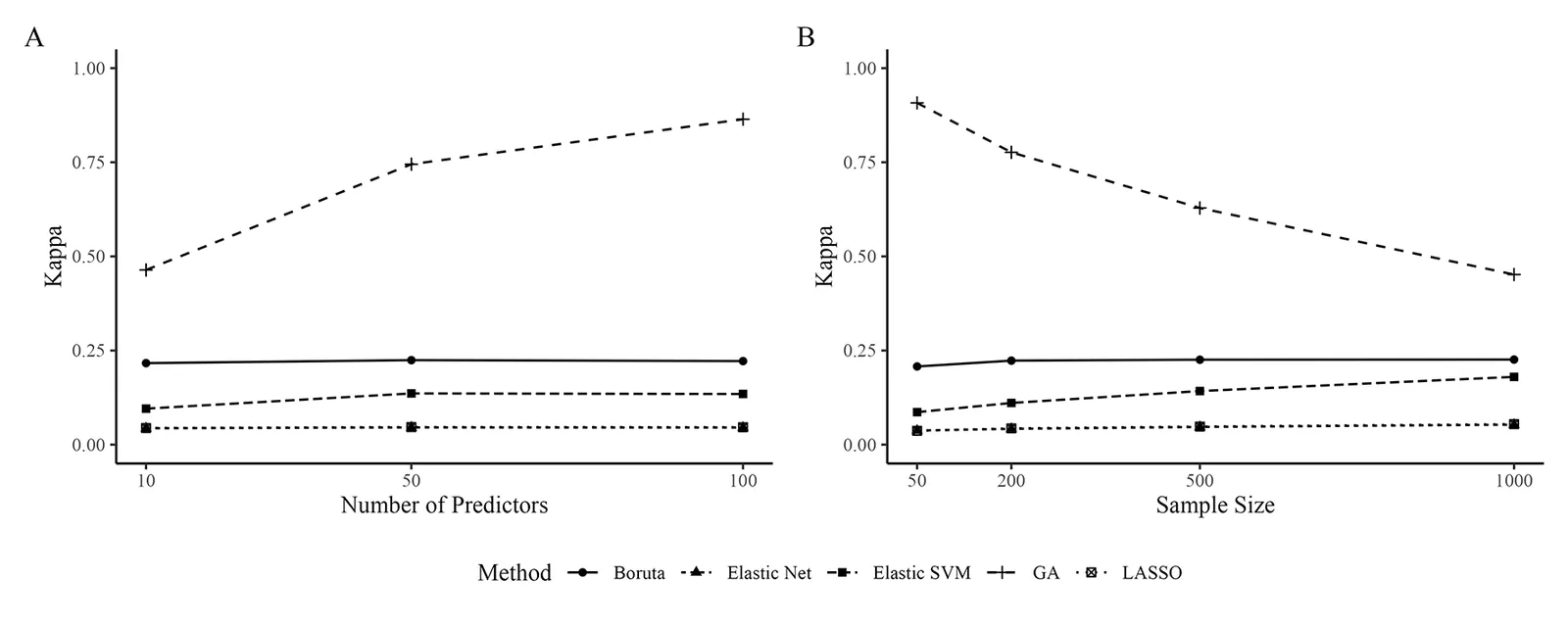
Comparison of kappa between methods across different conditions. Panel **(A)** displays the interaction of the number of predictors and the method on the Kappa. Panel **(B)** displays the interaction of the sample size and the method on the Kappa. The performance of LASSO and Elastic Net models is so similar that they overlap completely in both Panels **(A, B)**.

The number of predictors did not influence the performance of LASSO or EN models, but did influence the performance of Boruta, Elastic SVM, and GA models (Figure 6A; η^2^ = 0.06). Elastic SVM models saw increases in kappa as the number of predictors increased from 10 to 50, but no performance increases were seen when the number of predictors increased from 50 to 100. A similar relationship was observed for Boruta models, but the effect from 10 to 50 predictors was small. The influence of the number of predictors on kappa was greater for GA models, with the largest difference seen between 10 and 50 predictors. This indicates that the effect of the number of predictors on classification performance, at least when measured by kappa, is weaker the more predictors are present in the dataset. The sample size influenced the kappa of all models (Figure 6B; η^2^ = 0.07). The kappa of EN and LASSO models saw very slight increases as the sample size increased. The kappa of Boruta models slightly increased as the sample size increased from 50 to 200 people, but this effect then leveled off. Elastic SVM models showed sustained increases in kappa across all increases in sample size. Increases in sample size led to sustained decreases in kappa for GA models. However, GA models still maintained the highest average kappa values across all numbers of predictors and all sample sizes.

### Sensitivity and specificity

**Figure 7:**
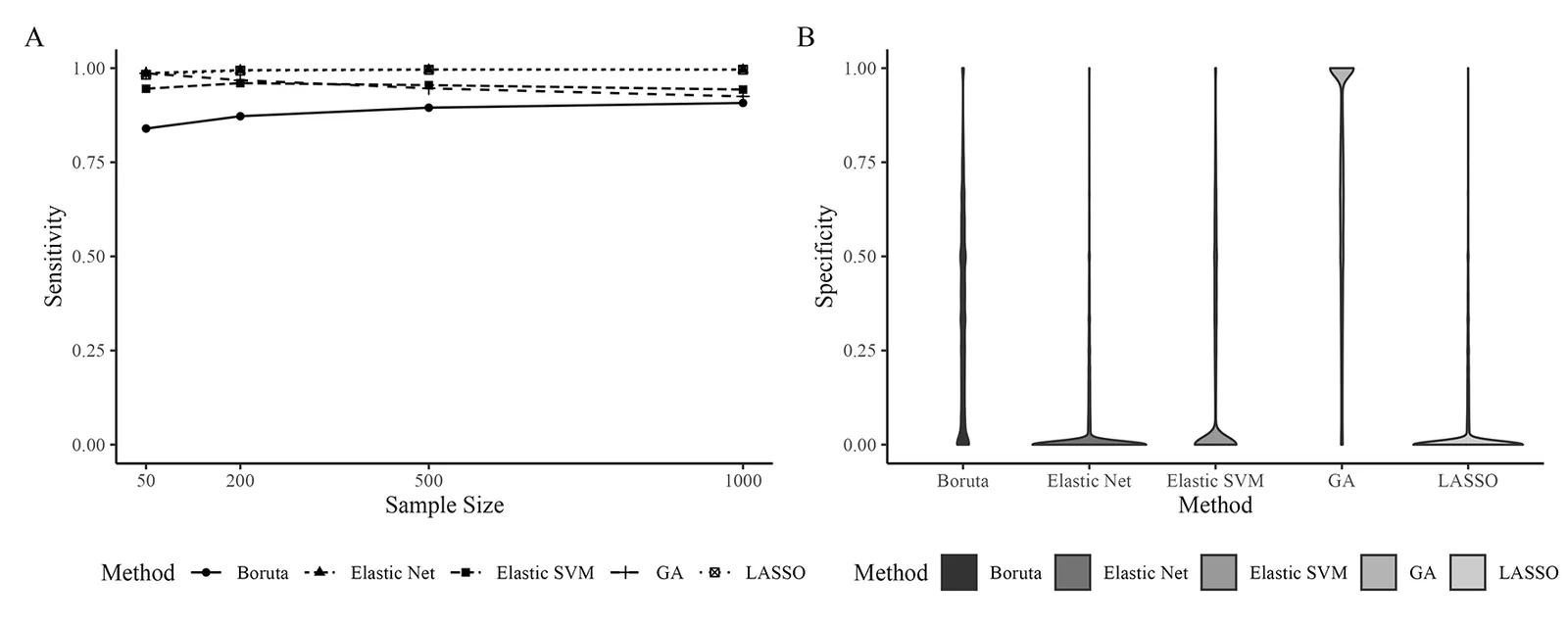
Comparison of sensitivity and specificity between methods across different conditions. Panel **(A)** displays the interaction of the sample size and the method on the sensitivity of models. Panel **(B)** displays the main effect of the method on the specificity of models. The performance of LASSO and Elastic Net models is so similar that they overlap completely on Panel **(A)**.

The sample size was found to be influential on the sensitivity of models (Figure 7A; η^2^ = 0.11). As the sample size increased, the sensitivity of Boruta, EN, and LASSO models also increased. Elastic SVM models saw better sensitivity values for sample sizes of 200 than 50, but as the sample size surpassed 200, the sensitivity decreased. The sensitivity of GA models decreased as the sample size increased. Specificity was found to differ across methods (Figure 7B; η^2^ = 0.43). GA produced models with the highest average specificity, while EN and LASSO models had the lowest average specificity. The specificity of all models, regardless of method, was highly variable.

### Effects of method choice on VS performance

#### Proportion of variables selected

**Figure 8:**
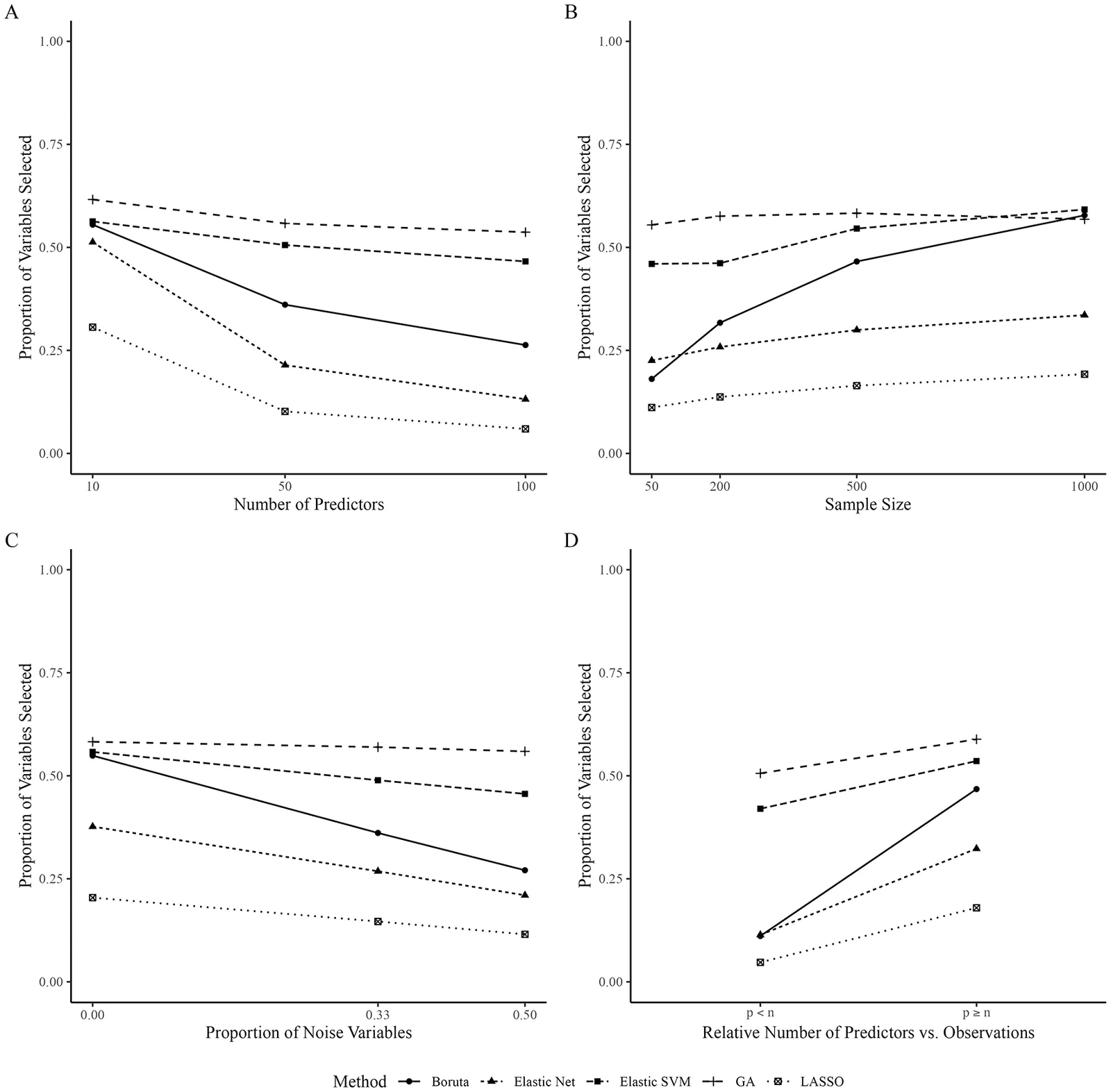
Comparison of the proportion of variables that were selected between methods across different conditions. Panel **(A)** displays the interaction of the number of predictors and the method on the proportion of variables selected in each model. Panel **(B)** displays the interaction of the sample size and the method on the proportion of variables selected in each model. Panel **(C)** displays the interaction of the amount of noise variables in the data on the proportion of variables selected in each model. Panel **(D)** displays the interaction of the relative number of predictors vs. observations and the method on the proportion of variables selected in each model. Unlike the classification-performance figures, LASSO and Elastic Net are distinguishable here: LASSO prunes more aggressively and therefore selected a smaller proportion of variables than Elastic Net across conditions.

The number of predictors was found to be influential on the proportion of variables selected by all methods (Figure 8A; η^2^ = 0.16). For all methods, as the number of predictors increased, the proportion of those variables that were selected decreased. This effect was strongest for LASSO and EN. The sample size was also found to be influential across all methods (Figure 8B; η^2^ = 0.21). For all methods but the GA, as the sample size increased, more variables were selected. For the GA, small reductions in the number of selected variables were seen as the sample size increased. The effect of sample size was strongest in Boruta models. The proportion of noise variables was also found to be influential for all methods, such that as more noise variables were present in the data, fewer variables were selected overall (Figure 8C; η^2^ = 0.08). This effect was weakest on the GA models, where the number of variables selected was fairly consistent and was strongest for Boruta models. All models selected fewer variables when there were fewer predictors than observations (*p*<*n*) than in other predictor to observation ratios (Figure 8D; η^2^ = 0.14). This effect was the strongest for Boruta models and weakest for GA, LASSO, and Elastic SVM models.

#### False positive rates

**Figure 9:**
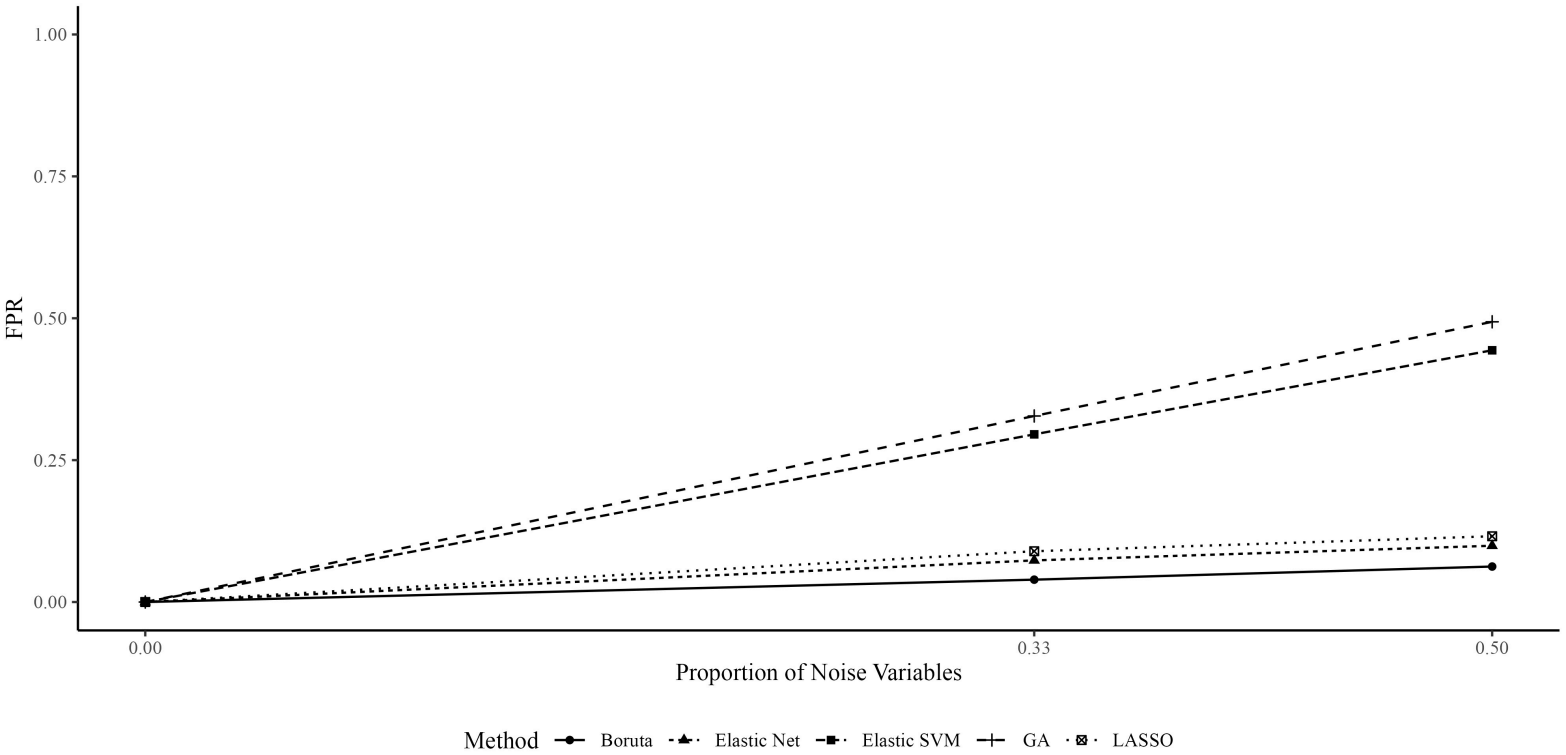
Comparison of the false positive rate of selected variables between methods across different conditions. FPR, false positive rate.

The false positive rates (e.g., the proportion of selected variables that were noise variables) were affected by both remove the proportion of noise variables, but this effect varied across methods (Figure 9; η^2^ = 0.40). As the proportion of noise variables increased, the FPR increased in all methods. This effect was strongest for GA and Elastic SVM methods and weakest for Boruta models. As a reminder, all ANOVA results for all outcomes are presented in Table 3.

#### Robustness under stratified 10-fold cross-validation

The trends reported above come from the single stratified hold-out split. To investigate if they are an artifact of one split, every method was re-evaluated under stratified 10-fold cross-validation using a subset of data conditions in supplementary simulations (full ANOVA results in Supplementary Table S4; Focused influential effect sizes can be found in Supplementary Table S5). The results from these simulations indicate two major findings. First, the influence of method on classification accuracy is weaker once evaluation is cross-validated: the method effect on AUC falls to a small effect (partial η^2^ = 0.03) from the large effect seen under the hold-out split (partial η^2^ = 0.30), indicating that some of the separation among methods narrows when performance is averaged across folds. Second, sample size was the strongest predictor of PER, AUC, Kappa, and specificity, while the prevalence rate was the most influential factor for sensitivity and predictor type was the most influencial factor for *F*1 score (Supplementary Table S5). The relative importance of these factors was not apparent in the hold-out results above. This suggests that some methods are more sensitive than others to whether performance is estimated from a single hold-out split or from cross validation.

Average performance was also investigated (Supplementary Table S1). All methods performed better with the continuous predictors than the binary predictors, which was not the case in the single hold-out sample where results were more mixed. Within data conditions with continuous predictors, LASSO had the best predictive accuracy according to PER (0.22), AUC (0.84), Kappa (0.45), specificity (0.84), and *F*1 score (0.73). However, eSVM, Elastic Net, GA, and the χ^2^ filter method outperformed LASSO in terms of sensitivity (μ_eSVM_ = 0.63, μ_EN_ = 0.62, μ_GA_ = 0.62, μ_χ2_ = 0.62, μ_LASSO_ = 0.61). This is notably different from the single hold-out results in which the GA approach had the best classification performance across all metrics, however, it is worth noting that the GA approach still performed well across all outcomes and was the only non-filter variable selection method that produced valid runs across all replications (Supplementary Table S1).

Lastly, we examined the difference in outcome measures between the train and test data (Supplementary Table S3). Boruta's classification performance was the most stable across train and test sets according to all classification metrics, while the GA was the least stable (Supplementary Table S3). For example, the difference between train and test AUC values for Boruta was only 0.06 while for GA it was 0.15. This indicates that the GA is likely to overfit the training data.

As a major purpose of this study was to compare across variable selection type, four filter methods (mutual information, variance threshold, Pearson correlation, and chi-square) were added during the supplemental simulations. Each filter scores predictors univariately, retains the top-ranked half, and passes them to a logistic classifier, so the filters share a common downstream model and differ only in the ranking criterion. Supplementary Table S2, summarizes their cross-validated performance alongside the embedded, wrapper, and metaheuristic methods.

Notably, the filters are competitive methods when examining AUC (all near 0.79) at a small fraction of the computational cost of the wrapper and metaheuristic methods, but because they retain a fixed half of the predictors by construction they are less parsimonious than LASSO, Elastic Net, and Boruta, which prune more aggressively. Filters therefore occupy a distinct corner of the accuracy, parsimony, and cost space: inexpensive and accurate, but not sparse.

#### The parsimony-accuracy trade-off

We were also interested in examining the nature of the relationship between parsimony and accuracy in the cross-validated supplementary simulations, however, as parsimony was fixed for the filter methods, they were excluded from this analysis. Supplementary Figure S2 reports loess models predicting test AUC by a parsimony value (calculated as 1—the proportion of variables selected) for each method. Most methods have a similar relationship within a narrow AUC band of roughly 0.72 to 0.86 across the full parsimony range. LASSO, Elastic Net, and Boruta show a modest, roughly monotonic decline in AUC as parsimony increases, indicating a mild accuracy cost for retaining fewer variables. E SVM behaves somewhat differently, holding a relatively stable AUC through mid-range parsimony before rising slightly at the most parsimonious end, suggesting it tolerates aggressive variable reduction better than the embedded methods. GA is the notable outlier: after tracking closely with the other methods at low parsimony, it dips to a local minimum near parsimony 0.45 to 0.5, then rises sharply and implausibly above an AUC of 1.0 beyond parsimony 0.6. Notably, this is an artifact of the spline model, as the GA approach has a maximum observed parsimony value of 0.64. It should not be interpreted as GA (logistic) achieving superior accuracy at high parsimony. Overall, this framing suggests that parsimony has only a weak influence on AUC for LASSO, Elastic Net, Boruta, and SVM.

## Empirical example

We apply the discussed VS techniques using an empirical example and compare their performance in classification. The dataset comes from a pre-existing dataset (Boness et al., 2019) comprised of 919 undergraduate students (${\bar{x}}_{age}=18.64$) at a large midwestern university. For simplicity, all methods were run on only complete cases, giving us a final sample size of 815. Most participants were female (56%) and White (91%). The dataset consists of 87 variables, 86 of which are AUD symptom indicators for the 11 DSM-5 criteria derived or adapted by Boness et al. (2019), and one of which is a binary item indicating a diagnosis of AUD according to the DSM-5 standards for moderate AUD or higher. All indicator items were rated on a five-point Likert scale (0 = Never/Not in the Past 12 Months, 4 = Yes, 4+ Times). Participants completed the entire survey battery online, and course credit was awarded upon completion. For our analyses, these items were then recoded to indicate the endorsement of a symptom (1) with a Likert rating of at least three or the absence of the symptom otherwise. The diagnosis variable was used for the pre-determined classification against which to check our models' predicted classifications.

To mirror the simulation framework in the applied setting, we broadened the empirical comparison in two ways. First, the four filter methods evaluated in the simulation (mutual information, variance threshold, Pearson correlation, and chi-square) were added to the applied comparison, each retaining the top half of the 86 candidate indicators and passing them to a logistic classifier, so that all four selection families (filter, embedded, wrapper, and metaheuristic) are represented on real data. Second, rather than relying on a single train and test split, every method was evaluated with stratified 10-fold cross-validation, and both training and cross-validated test metrics were recorded so that train-to-test gaps could be examined in the applied data as well. A consensus approach (where items are only considered selected if they were selected by all methods) was also discussed and the reliability and validity model formed via the consensus approach was reported.

### Results

Each of the methods was evaluated using several standard performance metrics: *F*1 Score, Prediction Error Rate (PER), Area Under the Receiver Operating Characteristic Curve (AUC), Cohen's Kappa, Sensitivity, Specificity, and the number of variables selected. We also noted the computation time required for each approach. These metrics were used to compare model effectiveness in classifying individuals with moderate or higher Alcohol Use Disorder (AUD) as defined by DSM-5 criteria.

### Classification performance

**Figure 10:**
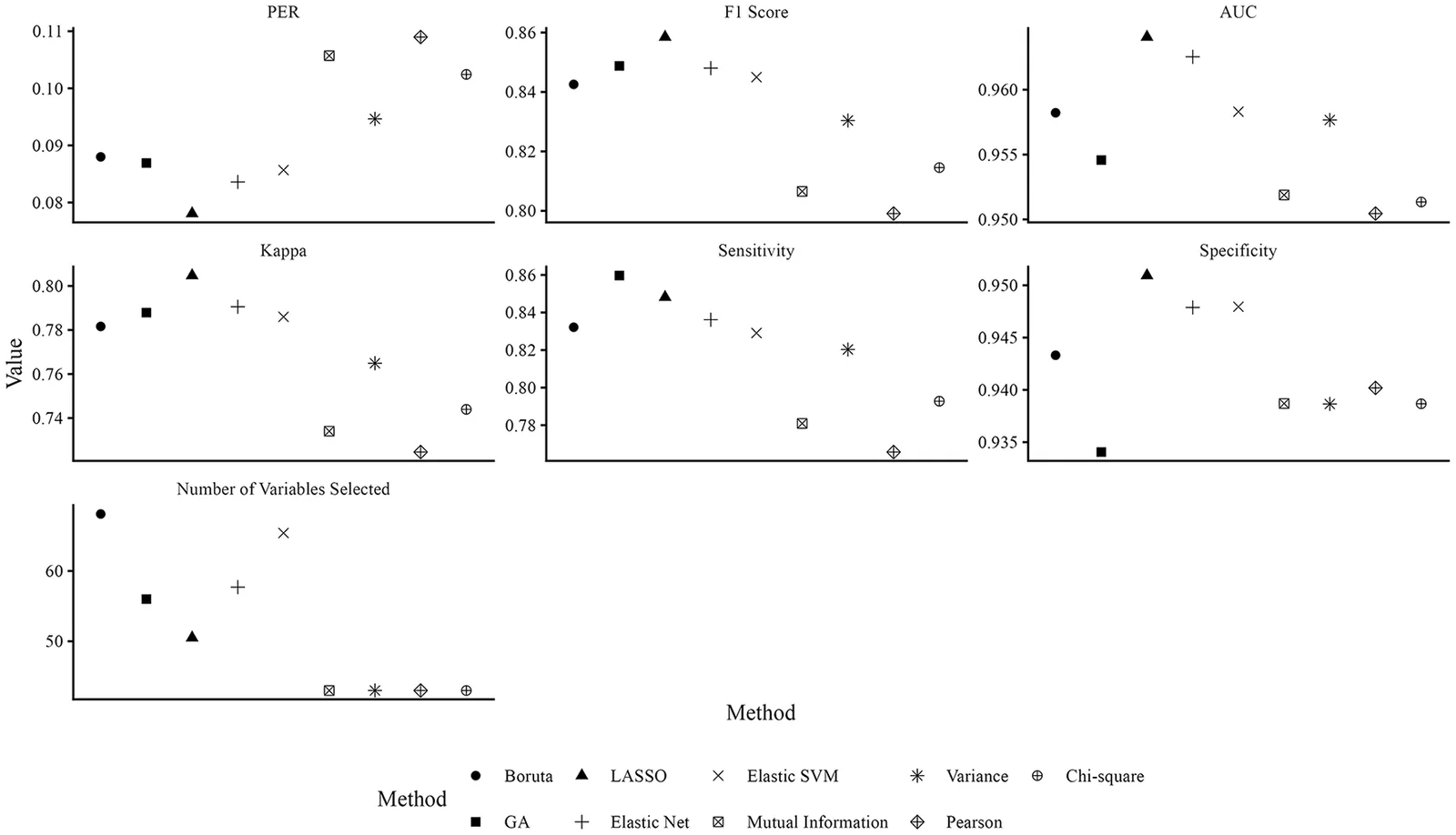
All outcomes for alcohol use disorder applied example.

Under stratified 10-fold cross-validation, every variable selection method produced strong classification performance, though performance varied by method (Figure 10 and Table 4). Among the methods, Boruta (*PER* = 0.09, *AUC* = 0.96, kappa = 0.78, *F*1 = 0.84), the GA (*PER* = 0.09, *AUC* = 0.95, kappa = 0.79, *F*1 = 0.85) and LASSO also performed well (*PER* = 0.08, *AUC* = 0.96, kappa = 0.80, *F*1 = 0.86). However LASSO selected the fewest variables (56), indicating that it might be a good choice for a balance between parsimony and accuracy. This attenuation of the GA advantage under cross-validation mirrors the overfitting pattern documented in the simulation, where the GA showed the largest train-to-test gaps (see Supplementary materials and the Overfitting analysis below). In contrast, Elastic SVM showed moderate performance despite selecting the second most variables (65.4), with only Boruta (68.1) selecting more.

**Table 4:** Applied AUD example: cross-validated outcomes and parsimony.

Method	# sel.	Test data						Train – test Δ					
		PER	AUC	Kappa	Sens.	Spec.	*F*1	PER	AUC	Kappa	Sens.	Spec.	*F*1
GA	56	0.09	0.95	0.79	0.86	0.93	0.85	−0.06	0.03	0.14	0.08	0.05	0.10
LASSO	50.5	0.08	0.96	0.80	0.85	0.95	0.86	−0.05	0.03	0.12	0.09	0.03	0.09
Elastic Net	57.7	0.08	0.96	0.79	0.84	0.95	0.85	−0.05	0.03	0.14	0.11	0.03	0.10
Boruta	65.4	0.09	0.96	0.79	0.83	0.95	0.84	−0.06	0.04	0.15	0.12	0.04	0.11
MI	68.1	0.09	0.96	0.78	0.83	0.94	0.84	−0.05	0.03	0.12	0.09	0.03	0.08
Variance	43	0.11	0.95	0.73	0.78	0.94	0.81	−0.03	0.02	0.07	0.06	0.02	0.05
Pearson corr.	43	0.09	0.96	0.76	0.82	0.94	0.83	−0.02	0.02	0.05	0.04	0.01	0.04
χ^2^	43	0.11	0.95	0.72	0.77	0.94	0.80	−0.03	0.02	0.07	0.07	0.01	0.05
Elastic SVM	43	0.10	0.95	0.74	0.79	0.94	0.81	−0.03	0.02	0.06	0.05	0.02	0.05

PER, prediction error rate; AUC, area under the curve; # selected = proportion of total variables that were selected; FPR could not be investigated, as it is impossible to tell what variables are “true” and what variables are “noise” in applied data. Metrics are means over held-out folds (stratified 10-fold CV). # selected is from the full-data fit; filters retain the top 50% by univariate score, then a logistic classifier evaluates the retained set.

The four filter methods provide a low-cost baseline against which the embedded, wrapper, and metaheuristic methods can be judged on real data. Because each filter retains a fixed half of the candidate indicators (43 of 86), they are not designed to produce parsimonious solutions, but they isolate the value of the ranking criterion itself. In the cross-validated applied results (Table 4), the filter methods achieved test AUCs of about 0.96–0.97 and kappa values of about 0.75–0.80, which was comparable to the regularized and metaheuristic methods while requiring a small fraction of their computation time. Among the filters, the variance threshold had the highest AUC which conflicts with the simulation results in which the univariate association filters slightly outperformed the variance threshold. This pattern reinforces the simulation conclusion that filters are an efficient screening tool but are not a substitute for methods that prune the predictor set.

### Variable selection

**Figure 11:**
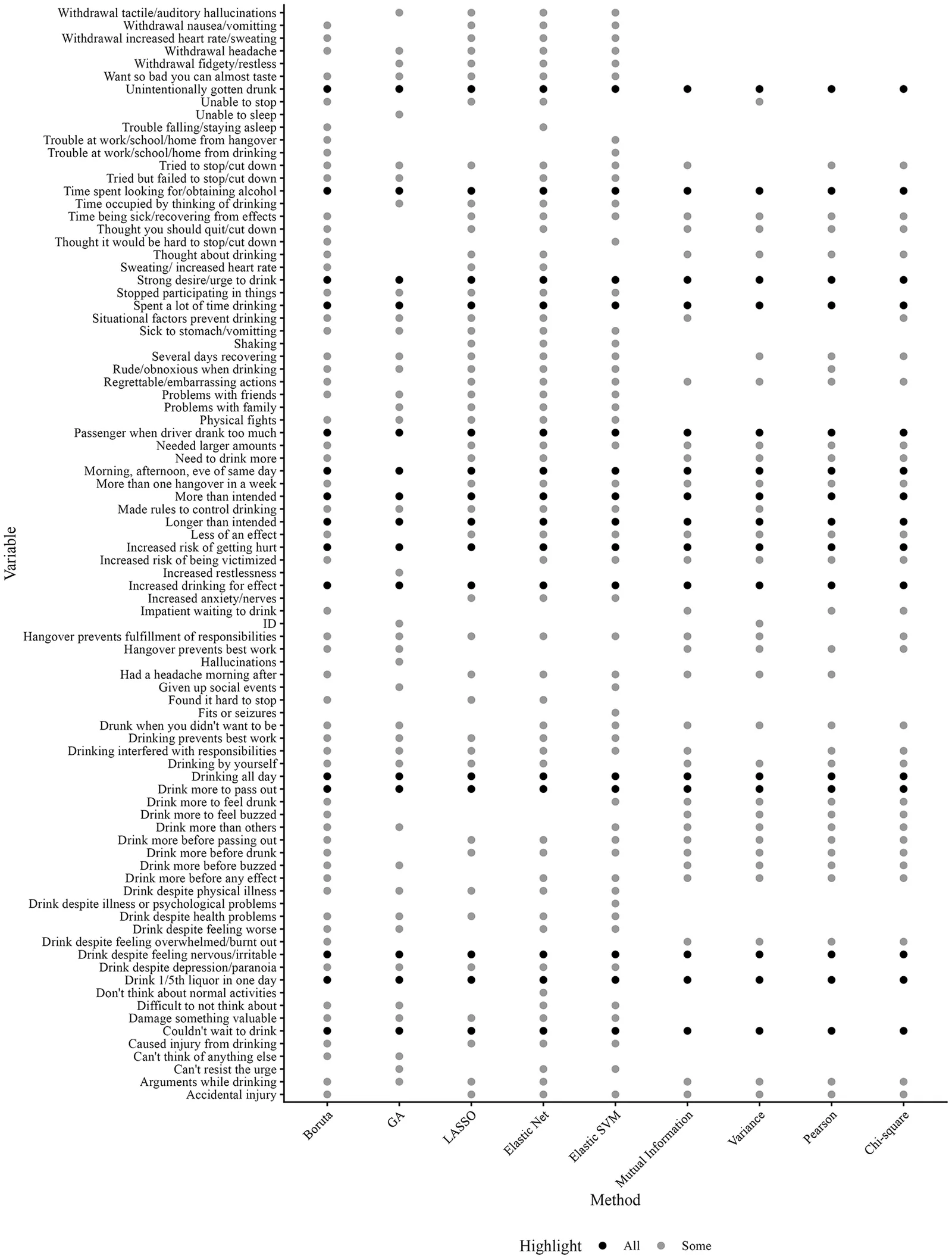
Variables selected for alcohol use disorder. Variables in gray were only selected by some methods, while variables in black were selected by all methods.

There were marked differences in the number of variables retained by each model. LASSO selected 50 variables on average and EN 57.7 variables on average, reflecting a marked decrease from the 86 candidate variables. The GA model retained 56 variables on average while remaining competitive in terms of classification metrics. Boruta selected 68.1 variables on average, while Elastic SVM, which also performed well, retained 65.4 variables on average. Which variables were selected by which methods can be found in Figure 11.

In terms of which variables were selected by each method, 15 variables were selected by all models (Figure 11), and 15 more variables were selected by all but one method. In addition, there were 5 variables that were only selected by one model, indicating they are likely not strong predictors of an alcohol use disorder diagnosis. Next, we examined the characteristics of the variables most often chosen by the techniques to potentially explain why they were selected.

Specifically, we examined the reliability of the measures created using a consensus approach, using just the Boruta, and using the full item set. We chose the Boruta model because it performed well but also selected a large number of variables. The full item set had 86 items (α = 0.955). Using Boruta, we had a scale with 71 items (α = 0.952). Using the consensus approach, we had a 15-item scale (α = 0.849). It is interesting to note that a scale with even only 15 items lead to an acceptable reliability, as one main goal of diagnosis for clinical purposes is to ensure a reliable scale. We also see that reliability is slightly better in the scale created using the Boruta approach than in the scale from the items not selected using the consensus approach. Also, the Boruta approach created a scale with only slightly worse reliability than the full item set. It is worth noting that, the 15 items for the consensus approach encompassed 7 of the 11 diagnostic criteria in the DSM-5. An endorsement of only six of the symptoms is required for severe AUD classification.

### Overfitting in the applied example

**Figure 12:**
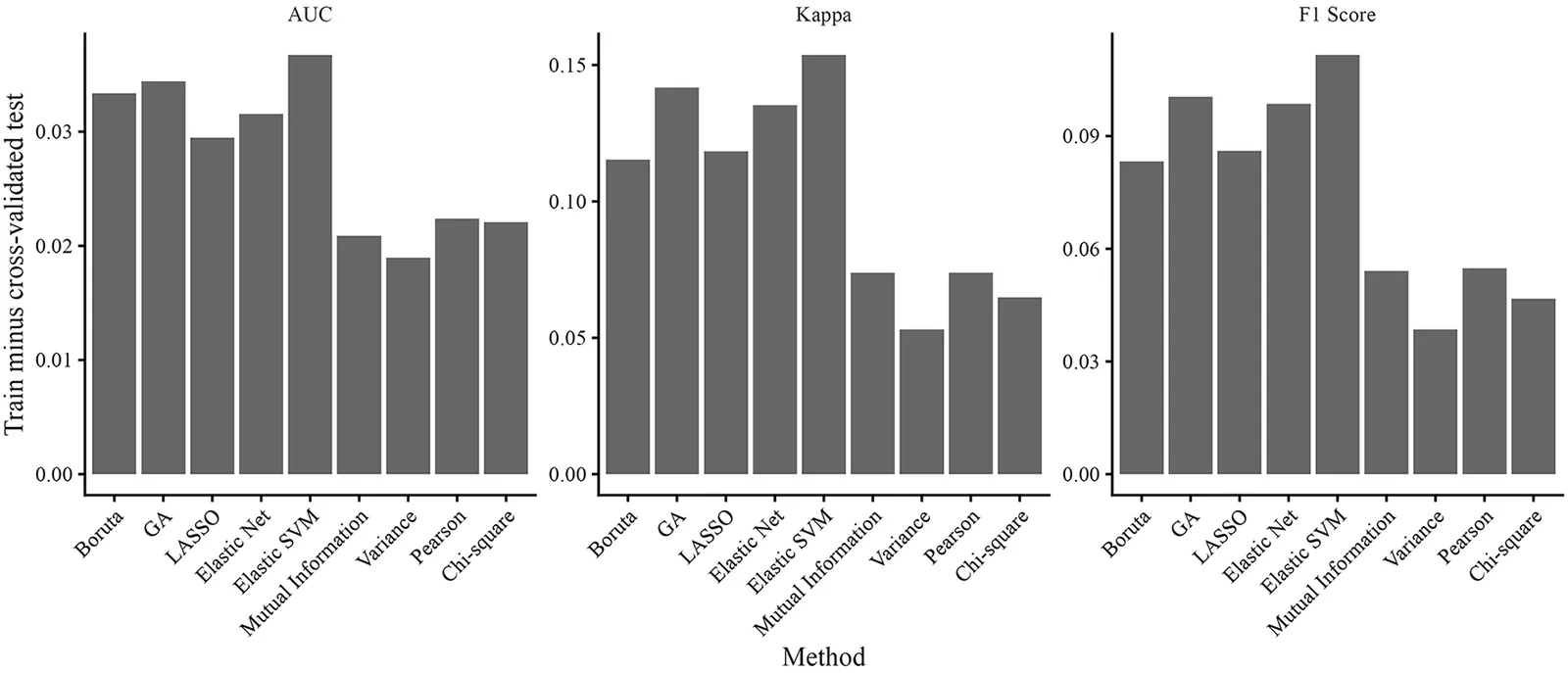
Training vs. cross-validated test performance for the alcohol use disorder applied example. For each method, the training metric and the cross-validated test metric are shown; the gap between them indicates the amount of overfitting that occurred in the training data such that smaller gaps are better.

Because both training and cross-validated test metrics were recorded, the train-to-test gaps observed in the simulation can be checked directly on the alcohol use disorder data. Figure 12 plots the training and cross-validated test values for each method, with the vertical distance between them representing the difference between the training and test validated set such that a smaller bar indicates a lower drop in performance from train to test. Notably, the filter methods had the smallest differences across AUC, Kappa, and *F*1 score. Elastic SVM had the largest, indicating it is most likely to overfit.

## Discussion

Through a large-scale Monte Carlo simulation and empirical validation using a dataset on alcohol use disorder (AUD), this study provided a comprehensive comparison of supervised machine learning VS methods commonly applied in the psychological sciences to two-group classification problems. By evaluating methods across classification performance, VS, and computational feasibility, this research offers pragmatic guidance for behavioral scientists tasked with selecting among diverse modeling strategies.

A key contribution of this study is the comparative evaluation of VS methods across methodological families (e.g., regularized regression, selection via variable importance measures, and metaheuristic approaches). With the additional four filter methods added in the supplemental simulations, the comparison now spans the full filter, embedded, wrapper, and metaheuristic taxonomy on both simulated and real data, allowing each family to be judged against the others.

In particular, the GA emerged as a top-performing method in the simulation under the single hold-out split, demonstrating the lowest PER, highest F1 score, and strongest kappa. In the cross-validated simulations, the GA did well, but was not the strongest performance. In addition, in the cross-validated empirical example, the GA remained competitive but did not outperform the Boruta wrapper approach (Table 4), aligning with the cross-validation simulation results. This is consistent with the overfitting analysis discussed below. Even so, the GA achieved this performance using substantially fewer variables than the full model. While the GA does require longer runtime than some methods, its computation time was well within practical bounds in our empirical example, especially given its competitive performance. It is worth noting that computation time may become unfeasible in other data conditions. The empirical findings also highlight that the GA performed well without having to hypertune GA parameters.

Two of the supplementary analyses, however, sharpen how this advantage should be interpreted. When every method was evaluated under stratified 10-fold cross-validation rather than a single hold-out split, the influence of method on classification accuracy was weaker. The rank ordering was preserved, with GA remaining among the strongest performers, but the magnitude of its separation from the regularized and filter methods was smaller. The overfitting analysis points in the same direction: GA showed the largest train-to-test gaps in AUC, indicating that part of its apparent superiority reflects its capacity to fit the training sample closely. Taken together, these results suggest that the GA advantage is best characterized as strong held-out performance obtained at the cost of the greatest optimism, rather than as a uniformly dominant solution. Researchers drawn to the GA for its accuracy should therefore evaluate it under cross-validation and report train-to-test gaps, rather than relying on a single split.

The results also affirm the use of LASSO and EN for researchers seeking highly interpretable models with minimal predictor sets. LASSO, in particular, selected the fewest variables, maintained strong predictive power, and executed quickly. These characteristics make it an ideal choice when simplicity, interpretability, and speed are prioritized. Grid-tuned variants did not improve on their cross-validated counterparts and incurred greater computational cost while selecting more predictors (see Supplemental materials). As such, cross-validation approaches available in standard packages like *glmnet* (Engebretsen and Bohlin, 2019) are recommended over grid search for these regularization methods.

Boruta also showed robust classification performance while substantially reducing the number of variables, placing it as a strong intermediate option between model parsimony and predictive performance. Notably, Boruta exhibited the smallest train-test difference of any method in the overfitting analysis (an AUC gap of 0.06 and an *F*1 gap of 0.05), which, together with its aggressive pruning, reinforces its position as a well-generalizing intermediate choice. Conversely, the Elastic SVM underperformed on all metrics, producing models with high error rates and low AUC. Additionally, the Elastic SVM model had a large computation time in our empirical example, making it an impractical choice in its current implementation using data within the scope of our simulation. Across all methods, VS was non-random and partially convergent. A subset of 15 variables was selected by all models in the empirical example.

The four filter methods occupy a distinct and practically useful niche. The outcome-aware filters (mutual information, Pearson correlation, and chi-square) performed well, within range of the GA and the regularized methods, while the variance threshold, which ignores the outcome, performed slightly worse. Because each filter retains a fixed proportion of predictors by construction, filters are not designed to yield sparse models and were the least parsimonious of the methods compared. Their appeal lies elsewhere: they are the least computationally demanding option by a wide margin and require no iterative search or penalty tuning. Filters are therefore best understood not as direct competitors to LASSO or the GA, but as an inexpensive first-stage screen that can trim a large candidate set before a more selective method is applied, a role that is especially attractive when the initial predictor pool is very large.

More broadly, the supplementary analyses reveal a consistent trade-off between model capacity and overfitting that is easy to overlook when methods are judged on a single hold-out split. Higher-capacity search procedures such as the GA achieve the best raw held-out accuracy but also overfit, whereas the regularized methods, the wrapper, and the filters give up a small amount of peak accuracy in exchange for narrower train-to-test gaps and, under cross-validation, performance much closer to that of the leading methods than the single-split results imply. For psychological applications, where replicability and out-of-sample estimates are paramount, this argues for routinely reporting cross-validated performance and train-to-test gaps alongside accuracy, and for weighting parsimony and stability, not accuracy alone, when choosing among methods.

Additionally, one limitation of the present design is that the GA was paired with a single internal classifier (logistic regression), so we cannot fully attribute its behavior to the search vs. the base learner within this study alone. A recent paper (Bain and Shi, 2026) addresses this by crossing SVM, k-nearest neighbors, and logistic regression as internal classifiers under a fixed GA search; it found that the internal classifier changed predictive performance and cost but not the selected variable set, which suggests the GA variable-selection results reported here are unlikely to be an artifact of the logistic base learner specifically. Extending the GA to non-linear internal learners (e.g., random forest or gradient-boosted trees such as XGBoost or LightGBM) within a single unified pipeline remains a valuable direction.

Together, these findings underscore the growing viability of machine learning-based VS in psychological research. Metaheuristic approaches like GA offer powerful, interpretable, and efficient alternatives to fully saturated models. Regularization-based methods provide accessible and fast options when model parsimony is crucial. Tree-based methods using variable importance metrics to select variables, such as Boruta, are well-suited for scenarios that balance interpretability with performance. Filter methods are recommended as a fast screening step rather than a stand-alone selection strategy. We advise against the use of Elastic SVM in its current form due to both poor classification performance and large computational demands. However, alternative implementations warrant exploration, as prior work has highlighted the potential of kernel-based methods under different data structures than were included in our simulation (Alonso-Atienza et al., 2012; Grigoryan, 2017; Javed et al., 2021; Orrù et al., 2012). Additional tree-based VS techniques may also be explored to broaden comparative evaluations (Speiser et al., 2019).

Several practical recommendations have also emerged from this study. Researchers with modest sample sizes and the need for strong classification accuracy should consider LASSO or GA models, depending on the tolerance for model complexity. Those seeking scalability or facing computational constraints may benefit from EN or Boruta, or a filter-based first-stage screen (see Supplementary materials). When reducing variable count is of the utmost importance, LASSO remains the leading choice. However, EN may be preferable in contexts where predictors are highly correlated, given its dual-penalty structure (Zou and Hastie, 2005). Furthermore, our findings emphasize the need for informed parameter tuning strategies. Default or cross-validated tuning typically suffices; extensive grid search often yields diminishing returns relative to the increase in computational burden. Just as importantly, the gap between the single-split and cross-validated results underscores that these recommendations should be based on cross-validated estimates: a ranking obtained from one hold-out split can overstate the advantage of the most flexible method, and reporting train-to-test gaps gives readers a direct check on that risk.

In conclusion, this study affirms that VS methods, particularly GA, LASSO, and EN, can match or exceed the performance of full-variable models while offering critical advantages in interpretability and efficiency. The supplementary analyses strengthen this conclusion: under cross-validation the leading methods remain strong while the differences among families narrow, the outcome-aware filters prove competitive at the lowest cost, and the train-to-test gaps clarify that the more parsimonious methods generalize with the least optimism. Their adoption promises to enhance replicability and model generalizability in psychological research, advancing methodological rigor in a data-rich era. Future research should continue exploring the integration of metaheuristic methods into hybrid models, the handling of missing data, and the applicability of Bayesian VS frameworks for added interpretability and precision (Bainter et al., 2023; Bondell and Reich, 2012; Shi et al., 2023).

## Data Availability

The datasets presented in this study can be found in online repositories. The names of the repository/repositories and accession number(s) can be found below: https://osf.io/qysh4/?view_only=85146f45202f4c9f944ee1d7622954b3.

## Footnotes

1 Users can choose to proceed with only confirmed variables or include tentative variables. For this paper, only those variables that were confirmed important were selected.

2 Note that the original Eisenbarth algorithm used *R*^2^ instead of ARI.
