# Supplementary material for: Comparing the use of supervised machine learning variable selection methods in the context of two-group classification in the psychological and health sciences
Source: Front Psychol. 2026 Sep 7;17:1819366. doi: 10.3389/fpsyg.2026.1819366 (PMC13590563; doi:10.3389/fpsyg.2026.1819366)

Supplementary Materials

Cross-Validated Re-Analysis with Filter Methods and an Overfitting Assessment

Companion to "Comparing the Use of Supervised Machine Learning Variable Selection Methods in the Context of Two-Group Classification in the Psychological and Health Sciences"

S1. Overview and Rationale

These supplementary simulations were conducted separately from the main study to address three points raised in review, without altering the main study's design or results. They (a) re-estimate method performance using stratified 10-fold cross-validation rather than a single hold-out split, (b) add a filter family (mutual information, variance threshold, Pearson correlation, and chi-square screening) to complete the filter–embedded–wrapper–metaheuristic comparison, and (c) compute training-set metrics and train–test performance differences so that overfitting can be compared directly across method families. Because they were run as a focused follow-up, they use a reduced but fully crossed condition grid; the design is described below and the exact grid is given in the accompanying conditions workbook.

The patterns reported here are consistent with the main study: cross-validation narrows the differences among methods relative to a single split; the higher-capacity methods show the largest train–test differences; and the outcome-aware filters are competitive with the embedded methods at the lowest computational cost. These analyses are intended as robustness and scope evidence supporting the main findings, not as a replacement for the main simulation.

S2. Methods

S2.1 Design

The supplementary grid fully crossed predictor type (binary, continuous), class prevalence (.20, .40), number of predictors (10, 100), noise level (none; an equal number of noise predictors), and sample size (50, 500), yielding 32 conditions. Each condition was replicated 100 times. All methods were evaluated within each replication using the same stratified 10-fold cross-validation partitions, so differences among methods are within-dataset differences.

S2.2 Methods compared

Nine selection procedures were evaluated: the genetic algorithm with a logistic base learner (GA), LASSO, Elastic Net (EN), the Boruta RF-based wrapper, and four filter methods — mutual information, variance threshold, Pearson correlation, and chi-square screening. Filters ranked candidates by their univariate association statistic and retained the top 50%, after which a logistic classifier evaluated the retained set, placing the filters on the same footing as the other families.

S2.3 Outcomes

For each replication we computed six classification metrics (prediction error rate [PER], AUC, Cohen's kappa, sensitivity, specificity, and F1) on the held-out folds and, in parallel, on the training folds. The train–test difference (training minus cross-validated test; Δ) was recorded for every metric as a direct index of optimism. Variable-selection outcomes were the proportion of variables selected and the false positive rate (proportion of selected variables that were noise).

Effect sizes are partial η^2 from factorial ANOVAs fit to the replication-level outcomes, using the same ≥ 0.06 (medium; Cohen, 2009) threshold as the main study.

S3. Results

S3.1 Cross-Validated Classification Performance

Under stratified 10-fold cross-validation, the method factor accounted for only a small share of variance in AUC (partial $\eta^2 = 0.03$), much smaller than in the single hold-out analysis, indicating that cross-validation substantially narrowed the differences among methods. On held-out folds, ranking by mean test AUC (Table S2), the methods ordered as LASSO > Elastic SVM and Boruta > Elastic Net > the outcome-aware filters (mutual information, Pearson, chi-square) > GA > the variance-threshold filter, spanning a narrow band from about 0.82 to 0.78, a narrower spread than under the single hold-out split. Prevalence rate remained the dominant data-characteristic effect (PER partial $\eta^2 = 0.10$; sensitivity partial $\eta^2 = 0.23$; F1 partial $\eta^2 = 0.08$). Full distributions by method and predictor type are in Table S1.

Table S1. Cross-validated test metrics by method and predictor type

Binary Predictors									
Method	N Invalid	PER	AUC	Kappa	Sens.	Spec.	F1	Prop Sel	FPR
GA (logistic)	0	0.32 (0.12)	0.76 (0.07)	0.27 (0.21)	0.53 (0.15)	0.74 (0.15)	0.61 (0.12)	0.63 (0.11)	0.23 (0.24)
LASSO	12	0.27 (0.11)	0.79 (0.08)	0.32 (0.23)	0.52 (0.21)	0.80 (0.12)	0.65 (0.12)	0.42 (0.29)	0.16 (0.19)
Elastic Net	6	0.28 (0.10)	0.79 (0.08)	0.31 (0.23)	0.52 (0.20)	0.79 (0.11)	0.64 (0.12)	0.50 (0.28)	0.18 (0.20)
Boruta	8	0.27 (0.10)	0.78 (0.08)	0.30 (0.23)	0.48 (0.22)	0.82 (0.12)	0.62 (0.14)	0.20 (0.19)	0.06 (0.15)
E SVM	9	0.29 (0.10)	0.78 (0.07)	0.31 (0.20)	0.54 (0.17)	0.78 (0.12)	0.64 (0.13)	0.40 (0.24)	0.16 (0.19)
MI	0	0.31 (0.12)	0.77 (0.07)	0.28 (0.21)	0.53 (0.16)	0.75 (0.15)	0.61 (0.12)	0.50 (0.00)	0.13 (0.16)
Variance	0	0.32 (0.11)	0.77 (0.07)	0.25 (0.20)	0.50 (0.17)	0.75 (0.15)	0.60 (0.14)	0.50 (0.00)	0.20 (0.20)
Pearson	0	0.31 (0.12)	0.77 (0.07)	0.28 (0.21)	0.53 (0.16)	0.75 (0.15)	0.61 (0.12)	0.50 (0.00)	0.13 (0.16)
Chi-sq	0	0.31 (0.12)	0.77 (0.07)	0.28 (0.21)	0.53 (0.16)	0.75 (0.15)	0.61 (0.12)	0.50 (0.00)	0.13 (0.16)
Continuous Predictors									
Method	N valid	PER	AUC	Kappa	Sens.	Spec.	F1	Prop Sel	FPR
GA (logistic)	0	0.27 (0.13)	0.82 (0.09)	0.39 (0.25)	0.62 (0.15)	0.77 (0.15)	0.68 (0.11)	0.58 (0.11)	0.24 (0.24)
LASSO	2	0.22 (0.11)	0.84 (0.08)	0.45 (0.25)	0.61 (0.20)	0.84 (0.10)	0.73 (0.10)	0.42 (0.28)	0.19 (0.22)
Elastic Net	2	0.24 (0.11)	0.83 (0.08)	0.43 (0.24)	0.62 (0.18)	0.82 (0.10)	0.72 (0.10)	0.55 (0.29)	0.21 (0.22)

Boruta	2	0.23 (0.10)	0.84 (0.07)	0.41 (0.23)	0.56 (0.20)	0.84 (0.10)	0.71 (0.11)	0.27 (0.25)	0.04 (0.11)
E SVM	0	0.23 (0.10)	0.84 (0.08)	0.45 (0.23)	0.63 (0.17)	0.82 (0.11)	0.72 (0.10)	0.41 (0.25)	0.14 (0.18)
MI	0	0.27 (0.14)	0.82 (0.09)	0.39 (0.26)	0.61 (0.17)	0.78 (0.16)	0.68 (0.12)	0.50 (0.00)	0.14 (0.17)
Variance	0	0.28 (0.14)	0.80 (0.09)	0.34 (0.26)	0.57 (0.19)	0.77 (0.16)	0.66 (0.13)	0.50 (0.00)	0.25 (0.26)
Pearson	0	0.27 (0.14)	0.82 (0.09)	0.39 (0.26)	0.61 (0.16)	0.78 (0.15)	0.68 (0.12)	0.50 (0.00)	0.12 (0.15)
Chi-sq	0	0.27 (0.14)	0.82 (0.09)	0.39 (0.26)	0.62 (0.17)	0.78 (0.16)	0.68 (0.12)	0.50 (0.00)	0.14 (0.17)

Note. Mean (SD) across replications on held-out folds. Higher AUC, kappa, sensitivity, specificity, and F1 are better; lower PER and FPR are better.

S3.2 Filter Methods vs. Other Families

The four filter methods performed similarly to one another and, on held-out folds, were broadly comparable to the embedded methods at the lowest computational cost. Mutual information, chi-square, and Pearson filters were nearly indistinguishable; the variance-threshold filter, which ignores the outcome, was weakest and retained the most noise (FPR = 0.22). Across the outcome-aware filters, mean held-out AUC was 0.79, mean kappa was 0.33, and mean PER was 0.29, compared with a LASSO held-out AUC of 0.81. These results support a two-stage workflow in which a cheap filter screens obvious noise before a multivariate selector (e.g., LASSO or GA) performs final selection.

Table S2. Filter methods vs. embedded, wrapper, and metaheuristic methods

Method	Test AUC	Test Kappa	Test PER	Prop Sel.	FPR	Δ Kappa
**Filter**						
MI	0.794	0.33	0.292	0.5	0.137	0.411
Variance	0.783	0.299	0.301	0.5	0.223	0.387
Pearson	0.794	0.332	0.291	0.5	0.125	0.412
Chi-sq	0.794	0.334	0.291	0.5	0.136	0.408
**Metaheuristic**						
GA (logistic)	0.791	0.33	0.295	0.606	0.237	0.506
**Regularization**						
LASSO	0.815	0.387	0.247	0.42	0.178	0.346
Elastic Net	0.808	0.373	0.258	0.523	0.196	0.394
E SVM	0.810	0.378	0.258	0.402	0.150	0.384
**Wrapper**						
Boruta	0.81	0.354	0.253	0.234	0.05	0.223

Note. Held-out test metrics and parsimony by method family.

S3.3 Overfitting: Train–Test Differences

Comparing training-fold and held-out performance revealed systematic differences in optimism across families. The higher-capacity methods showed the largest Δs: the GA exhibited the greatest difference (kappa Δ = 0.51; F1 Δ = 0.24), followed by the filter and embedded methods (e.g., LASSO kappa Δ = 0.35; EN kappa Δ = 0.39), while the RF-based Boruta wrapper showed the smallest Δ (AUC Δ = 0.06; F1 Δ = 0.05). The methods that looked strongest on training data were thus not always strongest out of sample, the comparison a test-only report cannot support. Ranked by kappa Δ, optimism was largest for the GA and smallest for the Boruta wrapper, with the filter and embedded methods in between. Figure S1 shows the full Δ distributions.

S3.4 Effect Sizes

Table S4 reports partial η^2 for the cross-validated analysis, including the filter methods in the Method factor and adding the train–test differences. For variable selection, the number of predictors (partial $\eta^2 = 0.51$) and sample size (partial $\eta^2 = 0.45$) influenced the proportion of variables selected, and the proportion of noise influenced the false positive rate (partial $\eta^2 = 0.80$). the Boruta wrapper produced the most parsimonious subsets (proportion selected approx. 0.23), while the GA retained the most among the non-filter methods (approx. 0.61); the filters retain a fixed fraction by construction.

Table S3. Training, cross-validated test, and train–test \Delta by method

Outcome	Method	Train	Test	Δ
AUC				
	GA (logistic)	0.94	0.79	0.15
	LASSO	0.93	0.82	0.11
	Elastic Net	0.94	0.81	0.13
	Boruta	0.87	0.81	0.06
	E SVM	0.93	0.81	0.12
	MI	0.93	0.79	0.13
	Variance	0.90	0.78	0.12
	Pearson	0.93	0.79	0.13
	Chi-sq	0.93	0.79	0.13
F1				
	GA (logistic)	0.89	0.64	0.24
	LASSO	0.82	0.66	0.13
	Elastic Net	0.84	0.66	0.16
	Boruta	0.71	0.64	0.05
	E SVM	0.84	0.68	0.16
	MI	0.82	0.64	0.18
	Variance	0.78	0.62	0.15
	Pearson	0.82	0.64	0.18
	Chi-sq	0.82	0.64	0.18
Kappa				
	GA (logistic)	0.84	0.33	0.51
	LASSO	0.73	0.39	0.35
	Elastic Net	0.77	0.37	0.39
	Boruta	0.58	0.35	0.22
	E SVM	0.76	0.38	0.38
	MI	0.74	0.33	0.41
	Variance	0.69	0.30	0.39
	Pearson	0.74	0.33	0.41
	Chi-sq	0.74	0.33	0.41
PER				

	GA (logistic)	0.07	0.29	-0.23
	LASSO	0.11	0.25	-0.14
	Elastic Net	0.10	0.26	-0.16
	Boruta	0.17	0.25	-0.09
	E SVM	0.10	0.26	-0.16
	MI	0.11	0.29	-0.19
	Variance	0.13	0.30	-0.17
	Pearson	0.11	0.29	-0.19
	Chi-sq	0.11	0.29	-0.18
Sensitivity				
	GA (logistic)	0.87	0.58	0.29
	LASSO	0.79	0.57	0.23
	Elastic Net	0.82	0.57	0.25
	Boruta	0.67	0.52	0.15
	E SVM	0.82	0.58	0.24
	MI	0.80	0.57	0.23
	Variance	0.75	0.54	0.21
	Pearson	0.80	0.57	0.23
	Chi-sq	0.80	0.57	0.23
Specificity				
	GA (logistic)	0.96	0.75	0.20
	LASSO	0.93	0.82	0.11
	Elastic Net	0.94	0.81	0.13
	Boruta	0.90	0.83	0.07
	E SVM	0.93	0.80	0.13
	MI	0.93	0.76	0.17
	Variance	0.93	0.76	0.16
	Pearson	0.93	0.76	0.17
	Chi-sq	0.93	0.76	0.17

Note. Δ = training metric minus cross-validated test metric; larger positive values indicate greater overfitting in the training data.

S3.5 Accuracy-Parsimony Tradeoff

Figure S2 reports loess models predicting AUC from the parsimony (calculated by 1 – the proportion of variables selected) by method. Most methods have a similar relationship within a narrow AUC band of roughly 0.72 to 0.86 across the full parsimony range. LASSO, Elastic Net, and Boruta show a modest, roughly monotonic decline in AUC as parsimony increases, indicating a mild accuracy cost for retaining fewer variables. E SVM behaves somewhat differently, holding a relatively stable AUC through mid-range parsimony before rising slightly at the most parsimonious end, suggesting it tolerates aggressive variable reduction better than the embedded methods. GA is the notable outlier: after tracking closely with the other methods at low parsimony, it dips to a local minimum near parsimony 0.45 to 0.5, then rises sharply and implausibly above an AUC of 1.0 beyond parsimony 0.6. Notably, this is an artifact of the spline model, as the GA approach has a maximum observed parsimony value of 0.64. It should not be interpreted as GA(logistic) achieving superior accuracy at high parsimony. Overall, this framing suggests that parsimony has only a weak influence on AUC for LASSO, Elastic Net, Boruta, and SVM, and that any claims about GA(logistic) at high parsimony levels should be treated cautiously pending inspection of the underlying data density and model fit in that region.

Table S4. All Effect sizes (partial η²) for the cross-validated analysis

Effect	PER	AUC	Kappa	Sensitivity	Specificity	F1	Prop. Selected	FPR
N	0.511	0.234	0.485	0.172	0.531	0.019	0.378	0.054
Method	0.072	0.026	0.029	0.021	0.121	0.048	0.59	0.298
# Preds	0.031	0.004	0.006	0.034	0.165	0	0.487	0.008
Noise	0.015	0.03	0.013	0.003	0.018	0.01	0.176	0.787
Type	0.092	0.125	0.123	0.093	0.042	0.145	0.004	0.003
Rate	0.104	0.01	0.038	0.231	0.263	0.074	0.007	0.001
N × Noise	0	0	0	0	0	0	0.02	0.054
N × # Preds	0.223	0.118	0.168	0.069	0.204	0.166	0.009	0.001
N × Type	0.014	0.04	0.023	0.021	0.002	0.058	0	0
N × Rate	0.001	0.03	0.001	0.007	0	0.234	0	0.001
Method × *N*	0.042	0.006	0.006	0.01	0.094	0.019	0.439	0.107
Method × Noise	0.004	0.006	0.005	0.004	0.002	0.004	0.164	0.298
Method × # Preds	0.06	0.019	0.016	0.014	0.125	0.023	0.465	0.003
Method × Predictor	0.002	0.005	0.001	0	0.002	0.002	0.033	0.017
Method × Rate	0.006	0.001	0.001	0.011	0.015	0.006	0.008	0.001
# Preds × Noise	0.001	0.004	0.003	0.004	0	0.004	0.013	0.008
# Preds × Rate	0.003	0	0	0.012	0.024	0	0.004	0
# Preds × Predictor	0	0.001	0	0	0.001	0.002	0.005	0.002
Predictor × Noise	0	0	0	0	0	0.001	0	0.003
Predictor × Rate	0	0.001	0.004	0.012	0.005	0.01	0	0
Rate × Noise	0	0	0	0	0	0	0	0.001

Note. Effect sizes < 0.06 (medium; Cohen, 2009) omitted. Includes the four filter methods. All outcome measures are the test values. Type = Predictor type; *N* = sample size; Rate = prevalence rate; # Preds = number of predictor variables.

Table S5. Influential Effect sizes (partial η²) for the cross-validated analysis

Effect	PER	AUC	Kappa	Sensitivity	Specificity	F1	Prop. Selected	FPR
N	0.511	0.234	0.485	0.172	0.531	–	0.378	–
Method	0.072	–	–	–	0.121	–	0.59	0.298
# Preds	–	–	–	–	0.165	–	0.487	–
Noise	–	–	–	–	–	–	0.176	0.787
Type	0.092	0.125	0.123	0.093	–	0.145	–	–
Rate	0.104	–	–	0.231	0.263	0.074	–	–
Method × *N*	–	–	–	–	0.094	–	0.439	0.107
Method × Noise	–	–	–	–	–	–	0.164	0.298
Method × # Preds	0.06	–	–	–	0.125	–	0.465	–
# Preds × *N*	0.223	0.118	0.168	0.069	0.204	0.166	–	–
Rate × *N*	–	–	–	–	–	0.234	–	–

Note. Effect sizes < 0.06 (medium; Cohen, 2009) omitted. Includes the four filter methods. All outcome measures are the test values. Type = Predictor type; *N* = sample size; Rate = prevalence rate; # Preds = number of predictor variables.

Figure S1. Train–test performance difference by method (AUC, kappa, F1)

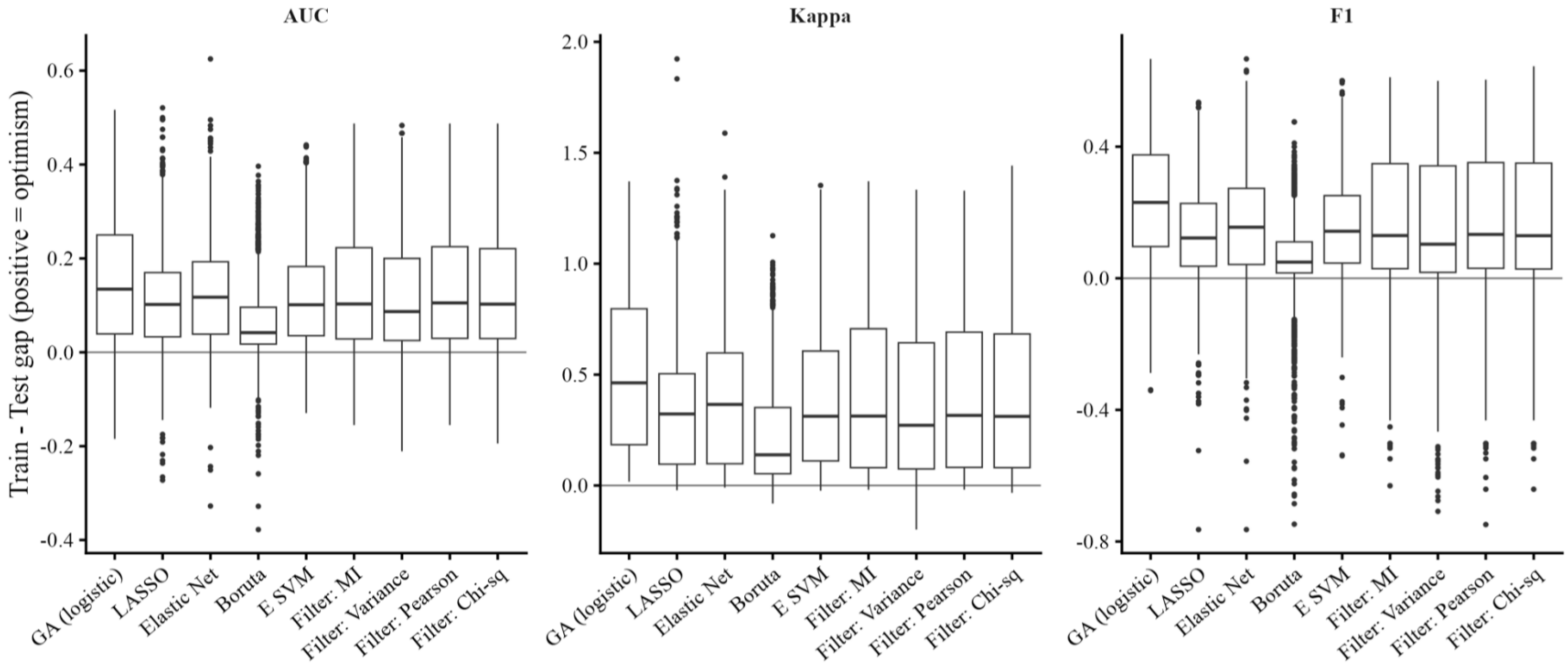

Figure S2. Balance Between Accuracy and Parsimony Across Methods

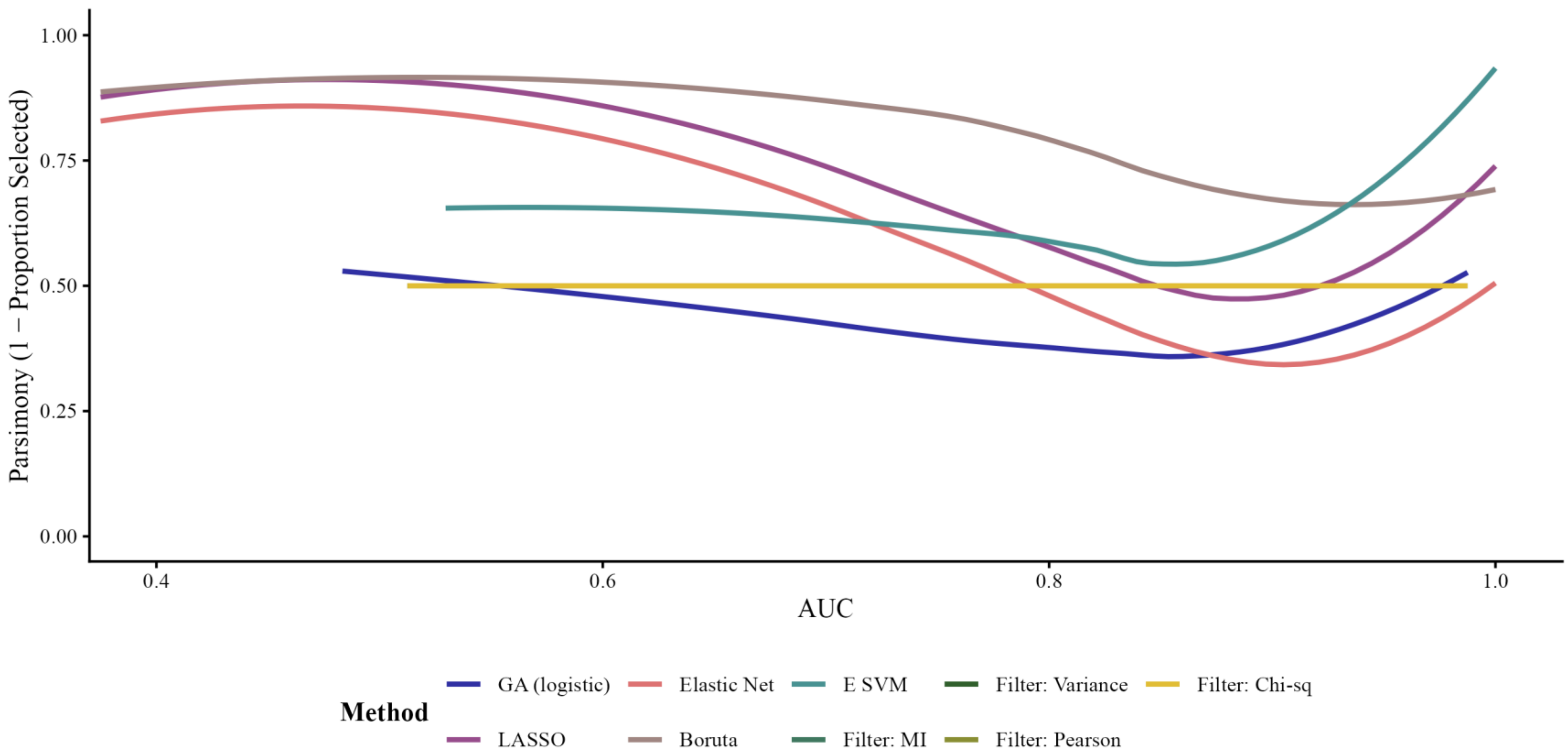

Supplement: Supplementary file 1 [file Data_Sheet_1.pdf]
